# An innovative method of evaluating the association of heat exposure and heart rate variability in a panel of agricultural workers with small and lightweight personal sensors

**DOI:** 10.1038/s41370-026-00848-9

**Published:** 2026-03-17

**Authors:** Shih-Chun Candice Lung, Shu-Chuan Hu, Cheng-You Tsai, Ming-Chien Mark Tsou, Jou-Chen Joy Yeh, Chun Hu Liu

**Affiliations:** 1 Research Center for Environmental Changes, Academia Sinica, Nangang, Taipei, Taiwan; 2 Department of Atmospheric Sciences, National Taiwan University, Taipei, Taiwan; 3 Institute of Environmental and Occupational Health Sciences, National Taiwan University, Taipei, Taiwan

**Keywords:** Farmers’ health, Heat and health, Occupational heat exposure, Personal sensors, Climate change and health adaptation, SDG 3

## Abstract

**Background:**

Agricultural workers are a highly exposed yet understudied group in the context of climate change.

**Objective:**

This study filled the research gaps in assessing personal heat exposure, its association with immediate changes in heart rate variability (HRV), and intervention choices for agricultural workers.

**Methods:**

Twenty-five participants were recruited from a pear orchard community, a rice seedling field, and a vegetable greenhouse farm in 2020 summer. Seven-day exposure of wet-bulb globe temperature (WBGT) was assessed for each participant with personal sensors for temperature, relative humidity, and solar radiation, as well as fixed-location monitoring for wind speed. These data were used as inputs for thermodynamic-principle-based Liljegren equations to calculate WBGT. Additionally, participants wore small medically certified HRV biosensors for 96 h.

**Results:**

WBGT levels were 27.0 ± 1.7, 31.9 ± 3.7, and 32.6 ± 6.3 °C for the participants at work in the pear orchards, rice fields, and greenhouse farm, respectively. The highest percentages above 32.2 °C of the rice-field and greenhouse participants were 60.8% and 66.3%, respectively. Peak WBGT reached 48.2 °C. The ambient WBGT from a fixed-location monitor underestimated the mean exposure, maximum exposure, and percentages above the critical thresholds of 32.2 °C and 36 °C for outdoor unshaded participants. The evident interpersonal variations emphasized the importance/necessity of individual WBGT assessments. A 1 °C WBGT increase was statistically significantly associated with a 1.05–2.44% decrease in all HRV indicators, except low-frequency/high-frequency, and a 0.65% increase in HR. Wearing gloves, masks, and long garments may aggravate HRV responses, whereas short garments may be protective.

**Significance:**

This is the first study applying personal environmental and biological sensors assessing WBGT and WBGT-HRV relationships of outdoor workers. The findings highlight the need for heat-health adaptation strategies, including personalized protective measures and WBGT-based warnings. The innovative methodology can be widely used for other outdoor workers.

**Figure:**
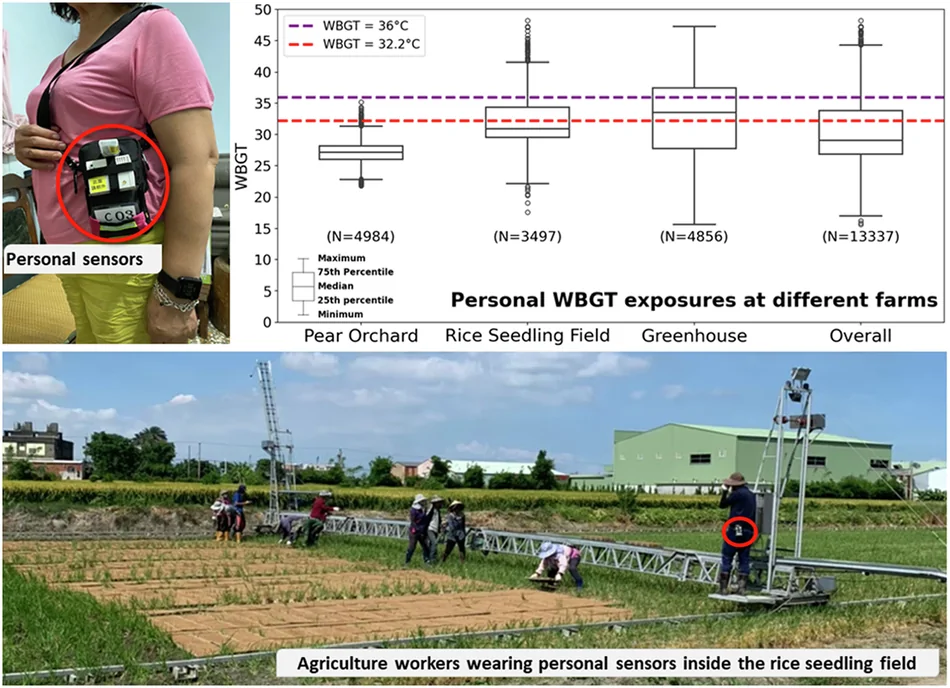

**Impact:**

This study addresses critical knowledge gaps in occupational heat exposure research by employing innovative exposure and epidemiological methodologies to assess personal WBGT exposure and its association with HRV in agricultural workers. The findings highlight substantial discrepancies between ambient and personal WBGT levels. By integrating advanced sensors and the OSHA-approved Liljegren equations to obtain WBGT, this study establishes a scalable framework for researchers and policymakers worldwide to accurately assess heat exposure, exposure–health relationships, and intervention strategies. These insights are particularly valuable for protecting agricultural workers as climate change intensifies occupational heat stress, thus fostering resilience in vulnerable populations and promoting overall health.

## Introduction

Heatwaves have caused significant casualties in recent years due to climate change [1]. In 2024, the global temperature was approximately 1.55 °C above pre-industrial levels [2]. Physiological responses aimed at maintaining homeostasis, such as increased heart rate and dilated blood vessels, have been shown to occur within minutes of heat exposure, indicating direct impacts on the circulatory system [3, 4]. Compared to healthy adults, individuals with chronic health conditions, including cardiovascular disease, hypertension, obesity, type 2 diabetes, and chronic kidney disease, often have impaired thermoregulatory functions and are therefore more vulnerable to the effects of extreme heat [4].

Outdoor workers, such as agricultural workers, are among the most vulnerable due to prolonged heat exposure [1]. It was shown that outdoor crop workers in the US die from heat stroke at a rate that is nearly 20 times higher than all other workers [5]. Additionally, a 20-year analysis in Taiwan identified farmers as a vulnerable group with the highest relative risk (RR) of cardiovascular emergency visits (RR = 1.12; confidence interval (CI): (1.08, 1.16)) among all occupational categories when the temperature was above 34 °C [6]. Surprisingly, very few publications focus on assessing actual heat exposure, measured by wet-bulb globe temperature (WBGT), and its association with health impacts for agricultural workers in the field, as is shown in the literature review below. The research gaps in heat exposure assessment, heat–health evaluations, and effect–modifier investigations hinder the development of effective adaptation strategies. Therefore, the current study targets this highly heat-exposed yet understudied group.

In Taiwan, the primary agricultural products include rice, vegetables, and fruits. As of 2020 (the year in which this study was conducted), the agricultural workforce numbered approximately 5,480,000, representing 4.8% of the total working population [7]. Among them, 74% were male, 45.2% were aged 50–64, and 19% were over 65, which is the most vulnerable population affected by heatwaves. Most agricultural workers (80.8%) were small-scale farmers working for family businesses. Those small-scale farmers may not have sufficient knowledge and resources on their own to combat the challenges brought by climate change.

To assess heat exposure, occupational agencies across the world have long used WBGT as a heat stress indicator [8, 9]. WBGT is also used in the newly proposed heat safety regulations of the Occupational Safety and Health Administration (OSHA), USA, in 2024 (https://perryweather.com/resources/osha-heat-safety-rules/). As a weighted combination of the dry-bulb temperature, natural wet-bulb temperature, and globe temperature, WBGT considers four heat-stress-related meteorological parameters: temperature, relative humidity, solar radiation, and wind speed. WBGT monitoring has traditionally been conducted with fixed-location monitors such as QuesTemp (3M^TM^, Oconomowoc, WI, USA) in workplaces, assuming uniform heat exposure levels across all workers. In a typical occupational monitoring campaign, one WBGT monitor was placed in one workplace to represent heat exposure of all workers in that workplace, sometimes even up to hundreds of workers. For example, one monitor was used to assess heat exposure of 517 sugarcane workers in Guatemala [10], 120 elderly farmers in South Korea [11], and 569 workers in El Salvador and Nicaragua [12]. Individual WBGT differences were ignored. In addition, outdoor workers often perform different tasks in various locations under the sun, and WBGT monitors cannot always be positioned nearby due to safety concerns or accessibility issues. This discrepancy between fixed-location WBGT monitoring and individuals’ actual WBGT exposure remains a scientific challenge, which leads to exposure misclassification. Moreover, the limitations of the non-waterproof and fixed-location design of these monitors, as well as the required daily maintenance to refill water for measurements, have restrained their applications. They are not suitable for long-term unmanned outdoor monitoring or personal monitoring.

Meanwhile, alternatives have been developed to calculate WBGT using meteorological parameters. Among them, Liljegren equations have been approved by the OSHA, USA, for WBGT estimation when direct measurements are unavailable [13]. Liljegren equations, developed by scientists at the US National Laboratory, are based on fundamental principles of heat and mass transfer [14]. Unlike other site-specific empirical equations [15], the thermodynamic-principle-based Liljegren equations are applicable across various locations, provided that meteorological inputs are available. Validation with WBGT measurements across seven US locations in 2007 showed a root mean square difference of less than 1 °C [14]. Another study obtained root mean square errors of 1.7 and 1.4 °C between Liljegren equations and the measured WBGTs in Georgia and Arizona, USA, respectively, in 2007 [16]. A more recent work reported WBGTs that were on average 0.2 °C higher with Liljegren equations compared with the observed WBGTs in multiple sites in North Carolina from 2019 to 2021 [17]. Some studies have applied Liljegren equations with meteorological data to evaluate heat stress on the scale of a city or region, such as Tokyo and South Asia [18, 19]. Nevertheless, no studies have used Liljegren equations to evaluate personal heat exposure.

Combined with Liljegren equations, recent advancements in sensor technology offer a promising solution for assessing personal WBGT exposure. This study pioneers the application of personal sensors to assess WBGT levels for individual outdoor workers. Up to January 2025, only twelve publications were found with the keywords of “WBGT,” “sensor,” and “exposure”; and only eight were found with “WBGT,” “sensor,” and “worker”, based on the Web of Science. Among them, only two studies applied personal sensors to measure WBGTs in the field. The first used a “heat stroke index” sensor (AD-5696, A&D Co., Ltd.) without disclosing the formula or theoretical basis for this measured WBGT [20]. The second study introduced a sensor-based safety helmet to assess WBGTs but only included sensors for temperature and humidity with a simplified formula, excluding solar radiation and wind speed [21]. Therefore, the current study is the first to use personal sensors measuring WBGTs in accordance with the original WBGT formula [13, equations shown in the Supplementary Materials].

Moreover, although over 7000 articles discuss climate change and heat impacts involving farmers’ health [22], only 55 publications were found with the keywords of “heat,” “farmer, agricultural worker, or agriculture worker,” and “WBGT”, based on the Web of Science in January 2025. Of these, only 23 conducted WBGT monitoring for agricultural workers in the field. Examples include assessments with different monitors for various outdoor farmers in Guatemala, South Korea, El Salvador, and Nicaragua [10–12, 23]. Only 14 of these 55 publications evaluated the association between the WBGT and health indicators such as heart rate (HR) and kidney function [12, 24, 25]. Notably, no studies have examined the associations between WBGT, considering all four input parameters, and heart rate variability (HRV) for agricultural workers. HRV is a marker of cardiac autonomic balance [26], with reduced HRV being associated with increased myocardial infarction risk [27]. Since heat stress directly impacts the circulatory system minutes after heat exposure [3, 4], it is important to assess the association of WBGT and HRV.

An individualized WBGT assessment is essential for small-scale farming, as agricultural workers often perform a variety of tasks across different shaded or unshaded outdoor microenvironments with varying levels of heat exposure. A detailed evaluation of specific tasks or microenvironments associated with high heat exposure can provide valuable insights for developing effective adaptation strategies in response to a warming climate. Additionally, individualized WBGT-health assessments can help raise awareness among workers, particularly those with pre-existing health conditions who may require special attention.

In light of these research gaps and urgent societal needs, the objectives of this study are to (1) assess the actual WBGT exposure of agricultural workers in Taiwan using personal sensors, (2) evaluate the association between the WBGT and HRV indicators reflecting immediate cardiovascular responses, and (3) explore potential intervention factors in the WBGT-HRV relationship. The findings can provide a scientific foundation for heat adaptation strategies to minimize the impacts of heat on the health of agricultural workers in Taiwan. The methodologies and lessons learned can be shared with the global scientific community to assess personal WBGT exposures and health impacts in high heat-exposure sectors across the world. Implementing adaptation strategies for agricultural workers aligns with the United Nations Sustainable Development Goal 3 (SDG3): ensuring healthy lives and promoting well-being for all ages [28].

## Methods

The following subsections describe the selected workplaces, participant recruitment, WBGT monitoring, heat–health evaluation, and data analysis in this panel study.

### Workplace description

Central and southern Taiwan are the main agricultural production areas in Taiwan. Three agricultural workplaces were selected in Central Taiwan: a pear orchard community, a rice seedling field, and a greenhouse farm. Their different business models may affect their ability to cope with challenges arising from climate change. The pear orchard community consists of farmers running their family orchards, handling everything from planting and harvesting to selling. The rice seedling field is operated mainly by a father-and-son team that hires temporary workers during intensive operation periods. The third workplace is a company-owned greenhouse farm with full-time employees cultivating leafy vegetables for export in high-tech and mechanically ventilated greenhouses made of transparent plastics. Greenhouse farming has been promoted due to its controlled conditions that protect crops from extreme weather and pests. The protocol for participant recruitment, interviews using questionnaires, WBGT exposure assessments, and health evaluations was reviewed and approved by the Institutional Review Board of Academia Sinica (No. AS-IRB-BM-20031). All methods were performed in accordance with the relevant guidelines and regulations.

### Participant recruitment

In Taiwan, COVID-19 had a relatively minor impact on daily life in 2020. Recruitment meetings were held in the three workplaces during the spring of 2020 to introduce the study’s objectives and methods. The eligibility criteria for participants were (1) being Taiwanese and aged 20–75, (2) no smoking and betel nut use for ≥1 year, (3) carrying out daily agricultural work ≥4 h, and (4) not drinking alcohol during work and having a daily intake <100 mL. Individuals with heart conditions or asthma, as well as those who stayed up for recreational purposes between 10 p.m. and 6 a.m., were excluded. Pre-existing health conditions, alcohol consumption, and irregular sleep schedules are known to affect the circulatory system [3, 4, 29], which could confound our primary focus on the effects of heat exposure. A panel of 25 participants was recruited, and written informed consent was obtained from all participants before joining the study. Trained interviewers conducted interviews to gather personal characteristics, working history, job and task descriptions, heat exposure experiences, and heat-reduction measures. The specific questions included outdoor working hours, perceptions of heat, heat-related illness experiences, water intake, and measures that potentially reduce heat exposures.

### Tasks and working conditions

#### Pear orchard community

The monitoring period covered the pear harvesting operation from June 30 to July 7, 2020. The pear harvesting season is from June to August. Pear handpicking is typically carried out early in the morning, minimizing sun exposure. After harvesting, pears are shipped out or stored in cold storage after being packaged indoors. Farmers usually began work around 4:30 and finished harvesting by 8:30. Tasks included handpicking pears, loading them onto transport vehicles (i.e., tricycles), driving to packaging areas, and returning for further harvesting. During handpicking, farmers were mostly shaded by the trees. After finishing handpicking, the farmers began the packing work, which took place indoors without air-conditioning to prevent the pears from being affected. Even with windows open and fans running, the indoor conditions in the room were stuffy. The main tasks indoors included unwrapping the paper bags for the pears, inspecting, and packaging pears in decorative plastic bags and into boxes. After packing, the farmers dried the used paper bags on the ground under the sun. After lunch, tasks included packing, drying, shipping, and delivery services, with most work being conducted indoors. Some farmers worked till 20:00 or even 23:00.

#### Rice seedling field

The monitoring period was from July 10 to July 18, 2020. The second rice-growing season occurs from mid-July to early August. This farm prepares seedlings for sale, involving medium-to-heavy labor outdoors without shelter. Indoor work occurs in a semi-open area for seed preparation, including mixing seeds with soil, pesticides, and fertilizers. The father-and-son team handled the initial seed treatment, while temporary workers managed subsequent tasks such as stacking seedling trays (weighing approximately 220–250 g per tray) and transplanting seedlings. The seedling trays were transferred to the rice paddy after sprouting, usually in the early morning or late afternoon to avoid strong sunlight; seedlings required daily irrigation, preferably before 9:00 or after 15:00. After transferring the trays, the workers had to cover the rice paddy fields with nets in the late afternoon to prevent birds from accessing them and remove the nets when the sunlight became strong the following day. When the seedlings were ready for sale, the workers rolled the seedlings into tubes, manually placed three tubes in one seedling tray, and then cleaned all the trays and equipment. Workers worked from 7:00 to 17:00 or 19:00, with lunch breaks in non-air-conditioned rooms with open windows.

#### Greenhouse

The monitoring period for the greenhouse farm, which comprises 11 large greenhouses, took place from July 13 to July 22, 2020. The temperature, humidity, and sunlight inside greenhouses were controlled by computerized panels, which adjusted shading nets, sprinklers, large electric fans at both ends, and rooftop panels. All employees were full-time workers with working hours from 7:30 to 17:00, including a lunch break (from noon to 13:30) in air-conditioned rooms. Tasks included soil tilling, watering, pest control, seed treatment, sowing, weeding, harvesting, storage, equipment maintenance, and administrative duties. Workers were exposed to humid conditions inside the greenhouse and sunlight while moving between greenhouses.

### Personal WBGT monitoring

This study used a hybrid approach to measure personal exposure, combining personal sensing, environmental monitoring, and Liljegren’s equations as described below. Personal WBGT exposure was assessed using meteorological parameter monitoring with personal sensors for each informed participant, with the data from these serving as inputs to the Liljegren equations to calculate the natural wet-bulb temperature and globe temperature [14, shown in the Supplemental Materials]. One-minute resolutions of temperature, relative humidity, and solar radiation were monitored using small and lightweight personal sensors. Each participant carried two HOBO sensors (UA and U12, Onset, Bourne, MA, USA): a UA sensor (5.5 × 3 × 1 cm, 20 g) measuring temperature and light intensity and a U12 sensor (7 × 5.7 × 2 cm, 50 g) measuring temperature and relative humidity. The percent coefficients of variation of 40 sets were 1.5% for temperature measured by the UA, 1.2% for temperature measured by the U12, and 2.8% for relative humidity measured by the U12, respectively (Fig. S1), indicating low variability across units. All sensors were cleaned before monitoring. Except during showering and sleeping (when sensors were placed outside the bathroom or on nightstands), participants carried both sensors at their waist or back for 7 days, with the UA sensor facing up; an example is shown in Fig. S2. Sensor placement was decided in discussion with the participants, who chose sensor positions that minimally affected their activities.

In addition, a UA sensor was co-located with the solar radiation sensor of an on-site fixed-location HOBO weather station (HOBO RX3003, Onset, Bourne, MA, USA) for comparison. A linear regression equation was developed accordingly to convert the light intensity readings from the personal sensors into solar radiation (Fig. S3a, *R*^2^ = 0.959). The percent coefficient of variation of 40 sets was 7% for light intensity (Fig. S3b); the slopes of regressions were not much different in fields in 2014 and 2020 (Fig. S3a, c, d). Thus, the linear regression obtained on-site in 2020 was used for converting light intensity into solar radiation.

In contrast, since personal wind speed exposure is difficult to measure, 1-min-resolution wind speed data were collected from the on-site fixed-location HOBO weather stations that were installed at each workplace. The weather stations were placed at ground level with sensors positioned 2 meters above the ground, meeting the following criteria: (1) being close to where most participants stayed or most tasks were performed, (2) being in an open area without blocking the wind or sunlight, (3) no heated surfaces and items being located in the surrounding, and (4) being on a stable surface without affecting the activities of the farmers.

During the 7-day monitoring period, participants completed time–activity diaries at 30-min intervals. These included activity details, indoor/outdoor status, ventilation conditions, garments, and heat-reduction measures. The time–activity diaries were reviewed daily by trained interviewers.

### Ambient WBGT monitoring

For comparison, ambient temperature, relative humidity, solar radiation, and wind speed data obtained from the on-site HOBO weather stations were used to calculate the ambient WBGT levels using Liljegren equations. The ambient WBGT levels during working hours were compared with personal WBGT levels at a 5 min resolution. The working hours for ambient WBGT monitoring in each workplace were defined as 7:00–12:00 and 13:30–18:59 for the pear orchards, 7:00–12:00 and 13:00–18:59 for the rice seedling field, and 7:30–12:00 and 13:30–16:59 for the greenhouse farm.

### Health evaluation

HRV readings were obtained using personal biosensors, RootiRx® (Rooti Labs Ltd., Taiwan), which is a water-resistant, portable, single-lead electrocardiogram event recorder and medically certified in the EU, US, and Taiwan (www.rooticare.com). It is a small (62 × 22.5 × 8.45 mm) and lightweight (14 ± 1 g) patch with a sampling rate of 500 Hz and has shown an average beat-per-minute correlation of 0.98 compared with standard 12-lead Holter monitors [30]. The RootiRx® devices were attached to participants’ chests for two 48-h sessions during WBGT monitoring. They were worn continuously, including during showering and sleeping. RootiRx® was used in our earlier works for healthy adults [31, 32]. This study used two time-domain indicators—the standard deviation of normal-to-normal intervals (SDNN) and the root mean square of successive differences between normal heartbeats (RMSSD)—as well as five frequency-domain indicators—the high frequency (HF, 0.15–0.4 Hz), low frequency (LF, 0.04–0.15 Hz), very low frequency (VLF, 0.003–0.04 Hz), total power (TP), and LF/HF ratio. The HR and activity levels of the participants were also measured. The SDNN has been recognized as a general indicator of overall autonomic nervous system activity. The LF/HF ratio is commonly interpreted as a measure of the balance between the sympathetic and parasympathetic nervous systems. Parasympathetic activity is primarily reflected in the RMSSD and HF, whereas sympathetic modulation is largely represented by the LF. The TP encompasses the spectral power across the ULF, VLF, LF, and HF bands, representing the overall variance in HR dynamics [33]. HRV parameters were typically averaged over 5 min to assess acute impacts, such as from heat exposure [26, 33–35].

### PM_2.5_ monitoring

Particulate matter with aerodynamic diameters ≤2.5 μm (PM_2.5_) was monitored due to its potential impact on HRV [31, 32]. One-minute PM_2.5_ data were collected using AS-LUNG devices (Academia Sinica-LUNG, Academia Sinica, Taiwan). One AS-LUNG-O set (outdoor type), powered by solar panels with PM sensors (PMS3003, Plantower, Beijing, China) inside a waterproof casing, was installed 3–5 meters away from the HOBO weather station. Eleven, four, and four AS-LUNG-I devices (indoor type) were deployed in the indoor environments of the pear orchards, rice seedling field, and greenhouse farm, respectively. Details on the AS-LUNG devices can be found in previous publications [36, 37]. PMS 3003 is a low-cost sensor. All PM_2.5_ devices were calibrated with research-grade instruments before deployment [38]. The intersensor variability of the 40 laboratory-tested AS-LUNG-O sets was 9 ± 6%. In addition, a field comparison was conducted for 10 AS-LUNG-O sets under temperatures of 25.9–40.9 °C and relative humidity levels of 43.4–93.8%, yielding an absolute percent difference of only 10 ± 9% between the calibrated sensors and a reference instrument [36]. Participants were assigned indoor or outdoor PM_2.5_ based on their time–activity diaries. For off-work periods, ambient PM_2.5_ data from the nearest monitoring station of the Ministry of the Environment, Taiwan, were used.

### WBGT calculation and categorization

Personal WBGT exposures were calculated using Liljegren equations with inputs from personal sensors (temperature, relative humidity, and solar radiation) and the on-site weather station (wind speed) based on outdoor and indoor WBGT formulas (Eqs. (1) and (2), respectively, in the Supplementary Materials). Based on our previous work, the indoor wind speed was assumed to be 0.3 m/s [39]. Outdoor and indoor WBGT levels were assigned to participants according to their locations in their time–activity diaries. Rain gauge readings from the HOBO weather stations determined rainy periods, which were removed from the dataset.

The average working hours for each participant were obtained based on their time–activity diaries. WBGT levels with durations shorter than half the average daily working hours were excluded. To match the HRV measurements, which were typically assessed in 5-min intervals, 5-min WBGT levels were calculated. That period was excluded if more than 1 min of data was missing within a 5-min window. All WBGT levels reported in this paper are at a 5-min resolution for consistency.

The newly proposed OSHA heat stress regulations for outdoor workers use a WBGT = 90 °F (32.2 °C) as a high heat trigger (https://perryweather.com/resources/osha-heat-safety-rules/), which was also used as the black-flag threshold in earlier regulations [40]. Additionally, in Taiwan, WBGT levels ≥32 °C and ≥36 °C are categorized as cautious and dangerous, respectively, by the Central Weather Administration [41], based on epidemiological evidence indicating statistically significant increases in emergency visits of heat-related illness of the general public in the entirety of Taiwan (RR = 1.49; CI = (1.44, 1.54)) when the WBGT exceeded 32 °C and in Taipei metropolitan area (RR = 2.66; CI = (1.70, 4.18)) when the WBGT exceeded 36 °C [42, 43]. Therefore, the percentages of WBGT levels exceeding 32.2 and 36 °C were calculated.

### WBGT-HRV models

For HRV indicators, to reduce any bias caused by artifacts, a noise filter was defined as a mean SDNN of >400 ms and a mean HR < 40 beats/min and >200 beats/min in a single 5-min segment, based on expert consultation with Rooti Labs Ltd. For healthy adults, an SDNN > 400 ms is substantially higher than the typical range of 20 and 70 ms, while a mean HR <40 beats/min or >200 beats/min is physiologically implausible for typical resting or occupational conditions [33, 44]. Observations during sleep were excluded, as physiological signals differ during sleeping and non-sleeping hours. All HRV indicators and HRs were log10-transformed to ensure normality.

Associations between the WBGT and HRV indicators were assessed using the generalized additive mixed model (GAMM) with a Gaussian distribution and an identity link function. The GAMM was chosen due to the inclusion of repeated measurements, correlations among observations from the same individual, and the nonlinearity of certain independent variables [45]. After log transformation, the skewness and kurtosis values indicated that those HRV indicators were very close to the Gaussian distribution (Table S1). Our model selection based on the Akaike Information Criterion is summarized in Table S2a. The final model retained the WBGT, PM_2.5_, age, gender, BMI, microenvironment, activity level, and time of day. The PM_2.5_ levels, which were adjusted in the models, are shown in Fig. S4. Additionally, sensitivity analyses were conducted by replacing PM_2.5_ with ozone in the models. Ambient ozone concentrations were obtained from the nearest air quality monitoring station, which is located more than 10 km from these workplaces. The results showed that the inclusion of ozone did not alter the associations between the WBGT and the HRV indicators. Thus, PM_2.5_, measured in the microenvironments where participants stayed, remained in the final models.

Dummy variables were used for age (≥40/<40 years), gender (male/female), BMI, and microenvironment (outdoor/indoor). BMIs were categorized into normal weight (<24 kg/m²) and overweight/obese (≥24 kg/m²) based on the definition of the Health Promotion Administration, Ministry of Health and Welfare, Taiwan. Activity level was defined as the average magnitude of activity intensity, calculated as $\sqrt{{X}^{2}+{Y}^{2}+{Z}^{2}}$, where *X*, *Y*, and *Z* are the maximum accelerations in the left–right, craniocaudal, and dorsoventral dimensions, respectively, as recorded by the RootiRx® devices.

To account for the correlation introduced by repeated measurements within participants, the GAMM was fitted with a random intercept for participant ID. In addition, the sensitivity analysis, including the workplace as a nested random effect, found the estimated variance to be near zero (2.77e−09), suggesting negligible clustering by workplace. To account for nonlinear fixed effects, the thin-plate spline function for activity level and the penalized cubic regression spline function for the time of day were also adjusted. Examples of nonlinear relationships between independent and dependent variables are shown in Fig. S5. Diagnostic checks of the smooth term for the time of day confirmed appropriate penalization, with an estimated k-index of 0.98 (*p* = 0.12), suggesting no underfitting or over-smoothing. A continuous first-order autoregressive correlation structure (corCAR) for the time of day was also included in the models. Data analysis was performed using RStudio Build 492 (RStudio, USA, 2022) and R version 4.2.1 (The R Foundation, Austria, 2022).

This study also aimed to evaluate potential interventions (i.e., effect modifiers of the WBGT-HRV relationship), such as garments worn by participants; thus, daily garments were recorded in the time–activity diaries and included in the intervention models. The garment dummy variables included bamboo hats, short sleeves, short pants/skirts, masks, gloves, and waterproof shoes. A second intervention model included long sleeves and long pants/skirts as separate variables, excluding short sleeves and short pants/skirts. Sun protection sleeves were not included due to limited use among participants. All other variables in these intervention models remained consistent with the main models (Table S2b).

## Results

### Participants’ characteristics, working conditions, and WBGT exposure

Eight, six, and eleven participants from the pear orchard community, rice seedling field, and greenhouse farm, respectively, participated in this study. Their basic characteristics are summarized in Table 1a. Of these 25 participants, there were more males and individuals under the age of 40 than in other groups. Their working years spanned a wide range, and most participants had been educated to above high school. Ten participants were classified as overweight or obese.

**Table 1:** Summary of (a) the characteristics of the 25 participants, (b) the working conditions, and (c) the heat-protection measures in the workplaces obtained from the interviews; numbers in the table are mostly the numbers of the participants who responded except those numbers in the age range (minimum and maximum ages), working years (mean ± standard deviation), and working year range (minimum and maximum years); overall percentages are shown in the last column.

		Pear orchards	Rice seedling field	Greenhouse	Overall
(a)		(8 participants)	(6 participants)	(11 participants)	(25 participants)
Sex	Male	4	5	10	19 (76.0%)
	Female	4	1	1	6 (24.0%)
Age	<40	1	4	10	15 (60.0%)
	≥40	7	2	1	10 (40.0%)
	Range	37, 66	23, 66	22, 53	22, 66
Education	High school or below	6	1	0	7 (28%)
	Above high school	2	5	11	18 (72%)
Working year	Mean ± SD	24.4 ± 17.5	14 ± 12.1	9.2 ± 7.3	15.2 ± 14.1
	Range	3, 53	1, 30	2, 30	1, 53
Body mass index	<24	5	4	6	15 (60%)
	≥24	3	2	5	10 (40%)
**(b)**					
Position	Employees	1	2	5	8 (32%)
	Employer	7	4	6	17 (68%)
Full-time or part-time	Full-time	8	3	11	22 (88%)
	Part-time	0	3	0	3 (12%)
Working days per week last month	1	0	0	0	0 (0%)
	2	1	0	0	1 (4%)
	3	0	0	0	0 (0%)
	4	0	0	0	0 (0%)
	5	0	1	11	12 (48%)
	6	1	2	0	3 (12%)
	7	6	3	0	9 (36%)
Feeling hot during work	Never	0	0	0	0 (0%)
	Seldom	0	0	0	0 (0%)
	Sometimes	0	3	1	4 (16%)
	Often	2	1	4	7 (28%)
	Always	6	2	6	14 (56%)
Outdoor working hours from 10 a.m. to 3 p.m.	0	6	0	0	6 (24%)
	<1 h	2	0	0	2 (8%)
	≥1 h, <2 h	0	0	0	0 (0%)
	≥2 h, <3 h	0	2	1	3 (12%)
	≥3 h, <4 h	0	2	10	12 (48%)
	≥4 h	0	2	0	2 (8%)
Heat sources encountered outdoors	Never	4	1	0	5 (20%)
	High-temperature soil	3	3	7	13 (52%)
	High-temperature machinery	2	2	11	15 (60%)
	Wood or charcoal heating	0	0	0	0 (0%)
	Gas or natural gas heating	0	0	0	0 (0%)
	Hot wood	1	0	3	4 (16%)
	Plastic sheet	0	1	8	9 (36%)
	Open-air burning	3	3	1	7 (28%)
How often do you encounter the above outdoor source?	Never	4	1	0	5 (20%)
	Less than once a month	1	1	0	2 (8%)
	1–3 times a month	0	0	0	0 (0%)
	1–2 times a week	1	2	0	3 (12%)
	3 or more times a week	2	2	11	15 (60%)
Heat sources encountered indoors	Never	6	3	0	9 (36%)
	High-temperature soil	0	2	7	9 (36%)
	High-temperature machinery	0	1	8	9 (36%)
	Wood or charcoal heating	0	0	0	0 (0%)
	Gas or natural gas heating	0	0	0	0 (0%)
	Hot wood	0	1	0	1 (4%)
	Plastic sheet	0	0	3	3 (12%)
	Open-air burning	1	0	1	2 (8%)
How often do you encounter the above indoor source?	Never	6	3	1	10 (40%)
	Less than once a month	0	0	0	0 (0%)
	1–3 times a month	1	0	0	1 (4%)
	1–2 times a week	0	0	2	2 (8%)
	3 or more times a week	1	3	8	12 (48%)
Feel physically uncomfortable due to hot weather while working	Never	0	0	1	1 (4%)
	Rarely	1	3	1	5 (20%)
	Sometimes	3	2	7	12 (48%)
	Often	0	0	1	1 (4%)
	Always	4	1	1	6 (24%)
Affecting work efficiency due to hot weather	Never	1	0	0	1 (4%)
	Rarely	1	3	0	4 (16%)
	Sometimes	1	2	8	11 (44%)
	Often	1	0	1	2 (8%)
	Always	4	1	2	7 (28%)
Have you ever experienced heatstroke, heat exhaustion, or heat cramps due to hot weather while working	No	3	5	6	14 (56%)
	Yes, but did not visit a doctor	3	1	3	7 (28%)
	Yes, and visited a doctor	2	0	2	4 (16%)
**(c)**					
Drinking water providers	Mostly provided by the employer	0	6	11	17 (68%)
	Mostly provided by yourself	8	0	0	8 (32%)
Quantity of drinking water while working (including breaks)	Up to 1.4 liters	0	0	0	0 (0%)
	1.5–2.1 liters	2	0	1	3 (12%)
	2.1–2.8 liters	6	1	2	9 (36%)
	2.8–3.5 liters	0	2	4	6 (24%)
	More than 3.5 liters	0	3	4	7 (28%)
Is there any of the following cooling equipment in the work area?	Sunshade	3	4	10	17 (68%)
	Electric fan	6	3	9	18 (72%)
	Air or water cooler	0	0	0	0 (0%)
	Hand fan	0	1	0	1 (4%)
	Air conditioner	0	0	3	3 (12%)
	Sprayer	0	0	0	0 (0%)
	None of the above (single choice)	1	2	1	4 (16%)
Apply sunscreen products while working	Do not use	7	5	8	20 (80%)
	Use sunscreen with a cooling effect	0	0	0	0 (0%)
	Use sunscreen without a cooling effect	1	1	3	5 (20%)
Where do you stay during the night	My residence	7	3	11	21 (84%)
	Employer-provided accommodation or a rented house	0	3	0	3 (12%)
	Relatives’ or friends’ home	1	0	0	1 (4%)
Do you enjoy air conditioning during the night?	No	3	3	1	7 (28%)
	Yes	5	3	10	18 (72%)

Table 1b summarizes the working conditions and perceptions of heat that were obtained from the interviews. Eighty-four percent (21/25) of the participants often or always felt hot at their workplaces. Between 10:00 and 15:00 on a typical working day, 56% (14/25) stayed outdoors for over 3 h. Additionally, 60% (15/25) and 48% (12/25) encountered heat sources during outdoor and indoor work three or more times per week, respectively, predominantly in the greenhouse farm. A total of 28% (7/27) often or always felt physically uncomfortable due to hot weather, and 36% (9/25) often or always felt that their work efficiency was affected by the hot weather. In addition, 44% (11/25) experienced heat-related illnesses (i.e., heat stroke, heat exhaustion, or heat cramps) during work, but only 16% (4/25) sought medical attention. These all indicate that more attention should be paid to agricultural workers to reduce their heat-related health risks. The percentages within each workplace are shown in Fig. S6a.

Table 1c summarizes protective measures against heat-related illness obtained from the interviews. Regarding hydration, all self-employed pear orchard participants drank 1.5–2.8 L of water. Most participants in the other two workplaces typically drank more than 2.8 L, which were provided by their employers, with 28% (7/25) drinking more than 3.5 L. Shade and electric fans were available in all three workplaces. Meanwhile, 28% (7/25) did not enjoy air-conditioning at night after a long day of hard work. The percentages within each workplace are shown in Fig. S6b.

The personal WBGT exposures of the 25 participants during different periods are summarized in Table 2a, including the entire monitoring period (excluding rainy periods), the non-sleeping period used for the evaluation of the WBGT-HRV association, and the working period, which is further divided into outdoor and indoor work. HRV indicators during the non-sleeping period, used to evaluate the WBGT-HRV relationship, are shown in Table 2b. All HRV indicators except LF/HF are higher in the greenhouse participants, presumably due to their younger ages. The temperature, relative humidity, solar radiation, and wind speed during the entire monitoring period, which were used for calculating personal WBGT exposure, are summarized in Table S3a. Besides, the ambient WBGT levels from the fixed-location weather station are listed in the last row of Table 2a; the four meteorological parameters that were used to calculate the ambient WBGT are summarized in Table S3b. The PM_2.5_ levels, which were adjusted in the WBGT-HRV models, are summarized in Table S3c.

**Table 2:** Summary of 5-min resolution of (a) personal wet-bulb globe temperature (WBGT) exposures during different periods (excluding rainy periods) calculated with Liljegren equations using inputs from personal sensors except wind speeds and (b) heart rate variability (HRV) indicators in the non-sleeping periods from RootiRx®.

(a)					
Personal exposures		Pear orchards (8 participants)	Rice seedling field (6 participants)	Greenhouse (11 participants)	Overall (25 participants)
WBGT (°C) (Entire monitoring period)	Mean ± SD	26.2 ± 2.1	28.4 ± 4.7	26.4 ± 5.9	26.8 ± 4.8
	Max	36.0	48.2	47.3	48.2
	Sample size	12,517	8778	17,609	38,904
WBGT (°C) (non-sleeping period)	Mean ± SD	26.6 ± 2.1	29.9 ± 4.1	27.9 ± 6.1	28.0 ± 5.0
	Max	36.0	48.2	47.3	48.2
	Sample size	5486	4682	10,529	20,679
WBGT (°C) (working period)	Mean ± SD	27.0 ± 1.7	31.9 ± 3.7	32.6 ± 6.3	30.4 ± 5.1
	Max	35.2	48.2	47.3	48.2
	Sample size	4984	3497	4856	13,337
WBGT (°C) (outdoor working period)	Mean ± SD	26.7 ± 2.5	32.9 ± 4.0	35.5 ± 4.7	32.9 ± 5.3
	Max	35.2	48.2	47.3	48.2
	Sample size	1460	2198	3440	7098
WBGT (°C) (indoor working period)	Mean ± SD	27.2 ± 1.2	29.6 ± 1.4	25.4 ± 3.2	27.1 ± 2.3
	Max	31.3	39.5	38.2	39.5
	Sample size	3422	894	1371	5687
WBGT (°C) (outdoor transportation during work, such as motorcycles)	Mean ± SD	26.4 ± 1.9	31.8 ± 2.4	36.8 ± 4.2	31.1 ± 3.6
	Max	30.4	40.1	42.9	42.9
	Sample size	102	396	39	537
WBGT (°C) (indoor transportation during work, such as trucks)	Mean ± SD	N/A	27.7 ± 1	28.7 ± 0.7	28.1 ± 1.0
	Max	N/A	28.8	29.4	29.4
	Sample size	0	9	6	15
Ambient WBGT (°C)^a^	Mean ± SD	30.0 ± 3.4	31.0 ± 2.2	32.3 ± 3.3	31.1 ± 2.9
	Max	41.9	41.2	43.1	43.1
	Sample size	893	1070	956	2919
**(b)**					
**HRV indices**	**Pear orchards 8 participants (sample size = 5486)**	**Rice seedling field 6 participants (sample size = 4682)**	**Greenhouse 11 participants (sample size = 10,529)**	**Overall 25 participants (sample size = 20,697)**	
SDNN	52.5 ± 29.5	49.8 ± 30.8	59.3 ± 26.3	55.3 ± 28.5	
RMSSD	22.6 ± 13.9	24.9 ± 16.7	28.2 ± 15.1	26.0 ± 15.4	
LF/HF	4.0 ± 3.1	3.8 ± 3.3	3.9 ± 3.0	3.9 ± 3.1	
LF	1250.6 ± 2221.5	1259.9 ± 1705.4	1455.6 ± 1482.4	1357.0 ± 1758.9	
HF	613.1 ± 1115.3	479.7 ± 713.7	587.3 ± 724.8	569.8 ± 845.4	
VLF	2549.1 ± 3866.9	2284.5 ± 3436.2	2928.8 ± 3083.0	2682.4 ± 3397.0	
TP	4508.5 ± 6624.6	4115.4 ± 5340.6	5056.1 ± 4772.4	4702.7 ± 5461.0	
HR	83.5 ± 11.8	94.1 ± 15.8	88.6 ± 16.3	88.5 ± 15.6	

^a^The ambient 5-min WBGT levels during the working period are presented, obtained from the measurements of the on-site fixed-location weather station serving as inputs to the Liljegren equations.

The overall WBGT exposure during working hours averaged 30.4 ± 5.1 °C, with 27.0 ± 1.7, 31.9 ± 3.7, and 32.6 ± 6.3 °C for the pear orchard, rice field, and greenhouse participants, respectively (Table 2a). The outdoor greenhouse participants experienced on average higher WBGT levels (35.5 ± 4.7 °C) than those in the other two workplaces; the same pattern occurred when the participants worked in outdoor transportation microenvironments such as trucks, with a mean of 36.8 ± 4.2 °C for greenhouse participants. The highest personal WBGT exposure recorded was 48.2 °C, and came from a participant in the rice seedling field. For the pear orchard participants, despite the high humidity (Table S3a) in the pear orchards’ indoor or shaded outdoor conditions, the personal WBGT levels experienced without solar radiation were much lower than the ambient WBGT levels. Being in the shaded versus unshaded microenvironments made a tremendous difference in the WBGT exposure. Notably, the ambient WBGT levels underestimated personal WBGT outdoor exposure by 1.9 °C ( = 32.9–31.0, rice seedling field) to 3.2 °C ( = 35.5–32.3, greenhouse) on average and by 4.2 °C ( = 47.3–43.1, greenhouse) to 7 °C (48.2–41.2, rice seedling field) at their maximums (Table 2a).

Table S4 lists the mean and maximum working and non-working hours across different classifications according to the participants’ time–activity diaries. Participants in the pear orchards worked the longest, averaging 9.5 h per day, with 29.7% being spent outdoors. The average working hours in the rice seedling and greenhouse farms were 8.8 and 7.5 h, with 71.1% and 72.3% being spent outdoors, respectively. WBGT exposure levels based on our classification are shown in Fig. S7. As expected, the WBGT levels during outdoor working hours were the highest among the different classifications, except for outdoor pear orchard participants in the shade.

**Fig. 1:**
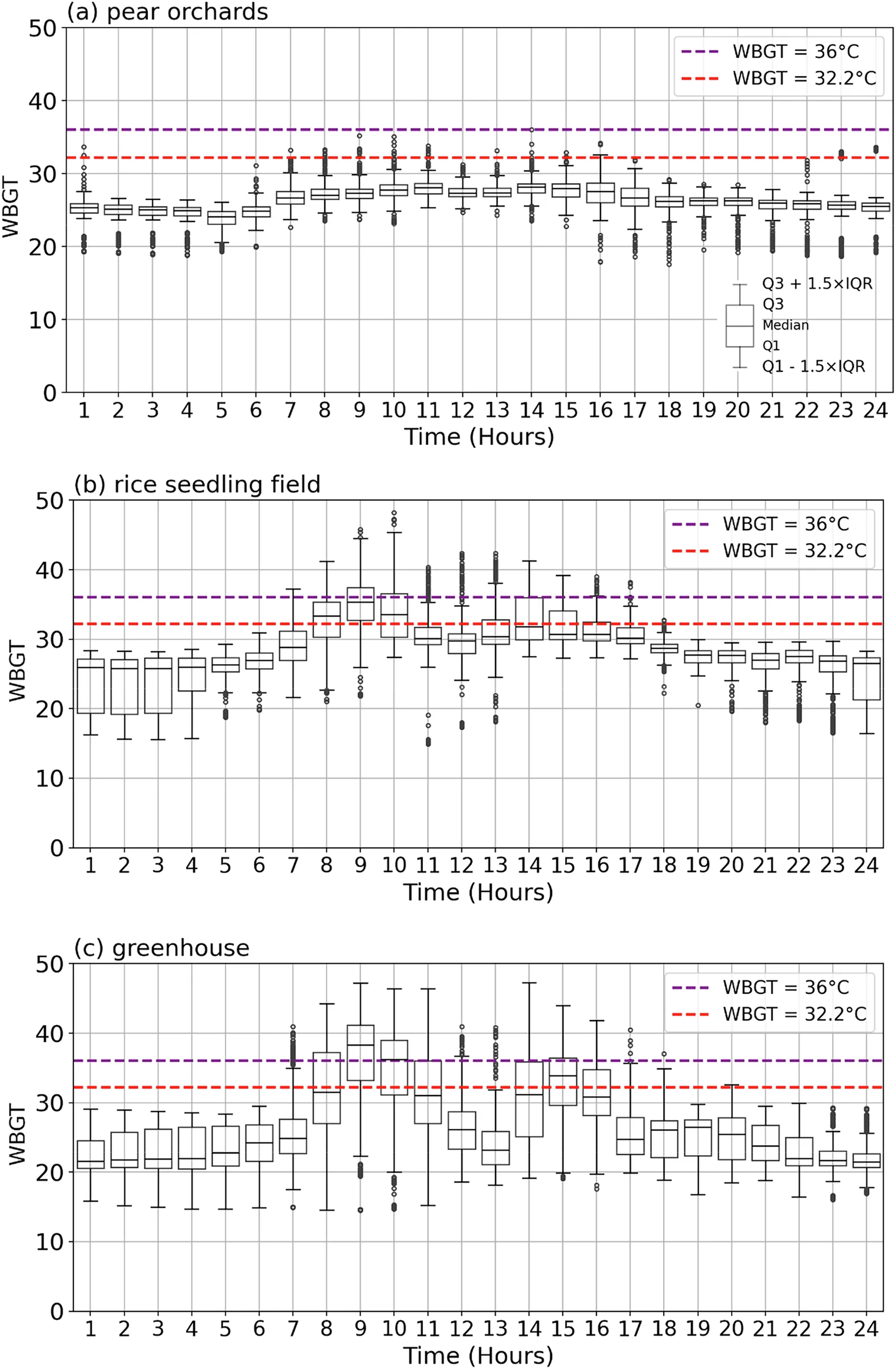
Personal WBGT exposure (°C, 5-min average) of all the participants in a diurnal cycle in the (**a)** pear orchard community (sample size = 12,517), (**b)** rice seedling field (sample size = 8778), and (**c)** greenhouse farm (sample size = 17,609). Q quartile, IQR interquartile range. Personal WBGT exposures were calculated using Liljegren equations with inputs from personal sensors (temperature, relative humidity, and solar radiation) and the on-site weather station (wind speed).

Figure 1 presents the WBGT exposure in a diurnal cycle for all participants. The diurnal temperature, relative humidity, solar radiation, and wind speed distributions are shown in Figs. S8–S11. The WBGT exposure of pear orchard participants between 7:00 and 16:59 remained similar, as they mostly stayed under tree shadows or indoors. In the rice seedling field and greenhouse farm, reduced WBGT exposure during lunch breaks was evident, with the highest median WBGT being registered around 9:00, not in the afternoon; these findings were consistent with the patterns of temperature and solar radiation (Figs. S8 and S10, respectively), with the highest medians being recorded at 9:00.

**Fig. 2:**
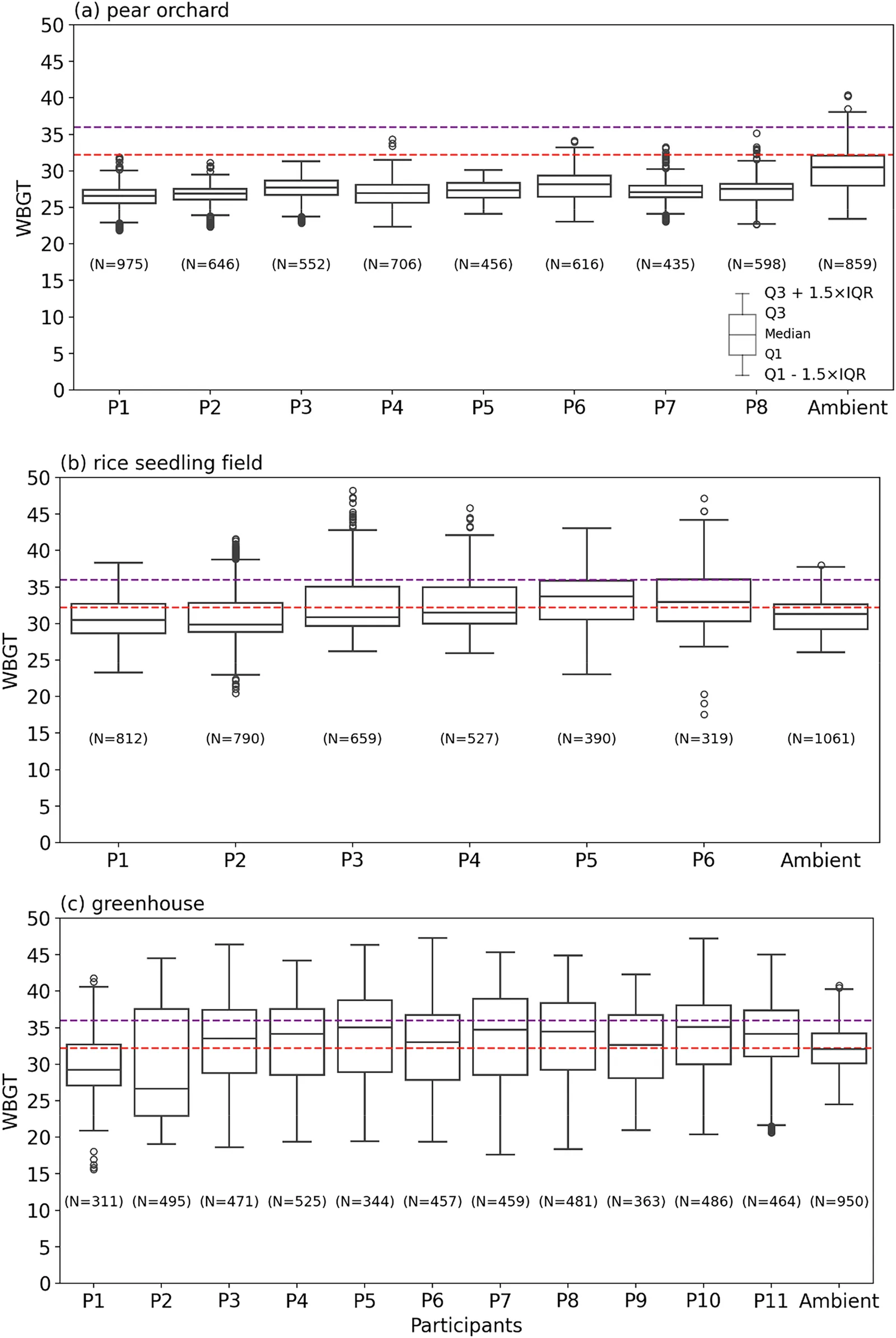
Personal WBGT exposure (°C, 5-min average) of individual participants during working hours in the (**a)** pear orchard community, (**b)** rice seedling field, and (**c)** greenhouse farm; the rightmost panels are ambient WBGT levels obtained from the fixed-location monitoring. P denotes each participant; N: sample size; Q quartile, IQR interquartile range.

Figure 2 shows the WBGT exposure levels for each participant during working hours. Variations in WBGT exposure among individuals in the same workplace, particularly regarding percentages above 32.2 °C (i.e., the OSHA-proposed heat stress threshold), emphasize the importance of individual WBGT assessments. The medians and percentages above 32.2 °C and 36 °C (the dangerous threshold in Taiwan [41]) of the ambient WBGT from the on-site fixed location weather station (the rightmost panel) were mostly lower than those of the personal WBGT levels in the rice seedling field and greenhouse farm.

The WBGT percentages for each participant and ambient levels above 32.2 °C and 36 °C are listed in Table S5a–c. The percentages of personal WBGTs exceeding 32.2 °C range from nearly 0% in the pear orchards to 27.2–60.8% in the rice seedling field and 27.3–66.3% in the greenhouse farm. The percentages of personal WBGTs above 36 °C range from 1.7 to 26.0% in the rice seedling field and 12.2 to 44.2% in the greenhouse farm, indicating severe heat exposure during working hours; meanwhile, the percentages of ambient WBGTs above 32.2 °C and 36 °C are 34.9% and 0.3% in the rice seedling field and 45.8% and 11.1% in the greenhouse farm, respectively. It is evident that the ambient WBGT measurements underestimated personal exposure for outdoor participants who were not in the shade.

Figure S12 shows the WBGTs during working hours based on gender, age, and BMI. Male participants and those under 40 had higher 25 percentiles, medians, and 75 percentiles of WBGT exposures than other groups. The percentages exceeding 32.2 °C and 36 °C (Table S5d) were higher for males, those under 40, and those with a BMI < 24.

### The association of WBGT exposure and HRV

In the main model (Table 3a), a 1 °C increase in WBGT is associated with a 1.05% (*p* < 0.001) to 2.44% (*p* < 0.001) decrease in all HRV indicators (except LF/HF) and a 0.65% (*p* < 0.001) increase in HR; the detailed *p* values are provided in Table S6a. Since reduced HRV has been linked to an increased risk of myocardial infarction [27], our results suggest potential cardiovascular impacts immediately following WBGT exposure. This further emphasizes the necessity of implementing heat adaptation strategies.

**Table 3:** The association of WBGT and heart rate variability (HRV) indicators in a 5-min resolution in (a) non-sleeping periods (excluding raining periods) (*n* = 24,786), (b) working hours considering clothing conditions excluding long sleeves and long pants (*n* = 9752), and (c) working hours considering clothing conditions excluding short sleeves and short pants (*n* = 9752).

(a)	SDNN	RMSSD	LF/HF	LF	HF	VLF	TP	HR
	(Adj *R*^2^ = 0.127)	(Adj *R*^2^ = 0.204)	(Adj *R*^2^ = 0.124)	(Adj *R*^2^ = 0.143)	(Adj *R*^2^ = 0.122)	(Adj *R*^2^ = 0.075)	(Adj *R*^2^ = 0.118)	(Adj *R*^2^ = 0.424)
WBGT	−1.13***	−1.05***	0.625***	−2.14***	−2.44***	−1.43***	−1.69***	0.647***
Age ≥40	−22.3*	−33.3**	5.92	−52.8**	−55.4*	−35.6	−42.5*	−2.95
Male	−2.80	−19.3	59.5**	−23.1	−51.9*	7.91	−12.8	−1.44
Overweight/obese	−10.8	−9.01	−13.4	−26.8	−15.6	−20.7	−20.5	1.34
PM_2.5_	−0.141	−0.185	0.340*	−0.138	−0.480	−0.309	−0.290	0.0374

(b)	SDNN	RMSSD	LF/HF	LF	HF	VLF	TP	HR
	(Adj *R*^2^ = 0.097)	(Adj *R*^2^ = 0.127)	(Adj *R*^2^ = 0.122)	(Adj *R*^2^ = 0.162)	(Adj *R*^2^ = 0.119)	(Adj *R*^2^ = 0.081)	(Adj *R*^2^ = 0.114)	(Adj *R*^2^ = 0.341)
WBGT	−0.742***	−0.849***	−0.413	−1.73***	−1.40***	−0.843**	−0.964***	0.612***
Short sleeves	3.49	2.69	3.22	14.0**	9.94	5.92	9.47*	−1.49*
Short pants (skirts)	7.12	2.68	12.0	1.74	−8.56	10.2	5.56	−5.19**
Gloves	−8.68***	−10.8**	3.52	−18.5**	−21.2**	−15.4**	−15.6**	0.995
Mask	−9.12***	1.70	−11.4**	−4.87	6.04	−23.3***	−12.9*	1.82
Waterproof shoes	−0.192	−2.33	−5.62	−6.31	1.19	1.79	0.763	3.01***
Bamboo hat	1.73	−0.859	−1.66	−1.90	−0.899	5.89	1.87	0.447
Age ≥40	−22.1*	−31.8*	−9.54	−56.8**	−52.2*	−34.2*	−41.8*	−2.58
Male	−15.2	−36.8*	75.6**	−50.4*	−71.5**	−12.9	−36.0	1.09
Overweight/obese	−3.63	−0.535	−15.7	−16.1	−1.19	−7.91	−8.26	−1.55
PM_2.5_	0.0316	−0.00863	0.0629	0.113	0.00188	0.0522	0.0863	−0.00184

(c)	SDNN	RMSSD	LF/HF	LF	HF	VLF	TP	HR
	(Adj *R*^2^ = 0.096)	(Adj *R*^2^ = 0.121)	(Adj *R*^2^ = 0.120)	(Adj *R*^2^ = 0.152)	(Adj *R*^2^ = 0.105)	(Adj *R*^2^ = 0.081)	(Adj *R*^2^ = 0.109)	(Adj *R*^2^ = 0.338)
WBGT	−0.763***	−0.862***	−0.436*	−1.78***	−1.45***	−0.883**	−1.01***	0.614***
Long sleeves	−4.36*	−5.05*	−1.46	−19.5***	−18.1***	−5.85	−11.8***	1.27
Long pants (skirts)	−5.44	−2.66	−9.07*	−3.94	5.31	−8.96	−5.92	3.95**
Gloves	−8.67**	−10.7**	3.51	−18.4**	−21.1**	−15.4**	−15.5**	0.849
Mask	−8.80***	2.07	−11.2**	−3.58	7.17	−22.9***	−12.2*	1.62
Waterproof shoes	0.263	−2.07	−5.13	−5.23	1.98	2.73	1.80	2.96***
Bamboo hat	2.56	0.0927	−1.40	1.97	2.74	7.11	4.23	0.268
Age ≥40	−21.0*	−30.9*	−8.86	−54.7**	−50.3	−33.1*	−40.1*	−3.10
Male	−15.0	−36.9*	76.7**	−50.6*	−71.8**	−12.7	−36.0	0.723
Overweight/obese	−4.24	−1.14	−16.1	−17.9	−2.99	−8.63	−9.47	−1.33
PM_2.5_	0.0239	−0.0114	0.0510	0.0988	−0.00343	0.0377	0.0711	−0.0007

The numbers in the table are percent changes of the respective HRV indicators for 1 °C of WBGT increase or 1 μg/m^3^ of PM_2.5_ increase; age ≥40, male, and overweight/obese were set as 1 in the age, gender, and BMI categories, respectively.

*Adj R*^*2*^ adjusted *R*^2^.

*: 0.01 ≦ *p* < 0.5; **: 0.001 ≦ *p* < 0.01; ***: *p* < 0.001, the detailed *p* values are listed in Table S4.

Internal heat generation due to workload results in additional physiological heat stress [46]. The participants’ workloads were represented by their activity levels, measured by the biosensors, which were adjusted in the WBGT-HRV evaluation. Figure S13 illustrates the activity levels of our participants over a diurnal cycle, with notably higher activity levels during daytime working hours.

Age, gender, and BMI are important determinants of HRV [4, 33, 35, 47]. In our analysis, elderly and male participants were associated with a more substantial decrease in certain HRV indicators in the intervention models, while BMI was not a statistically significant factor. This may be due to the small sample size of only 25 participants.

**Fig. 3:**
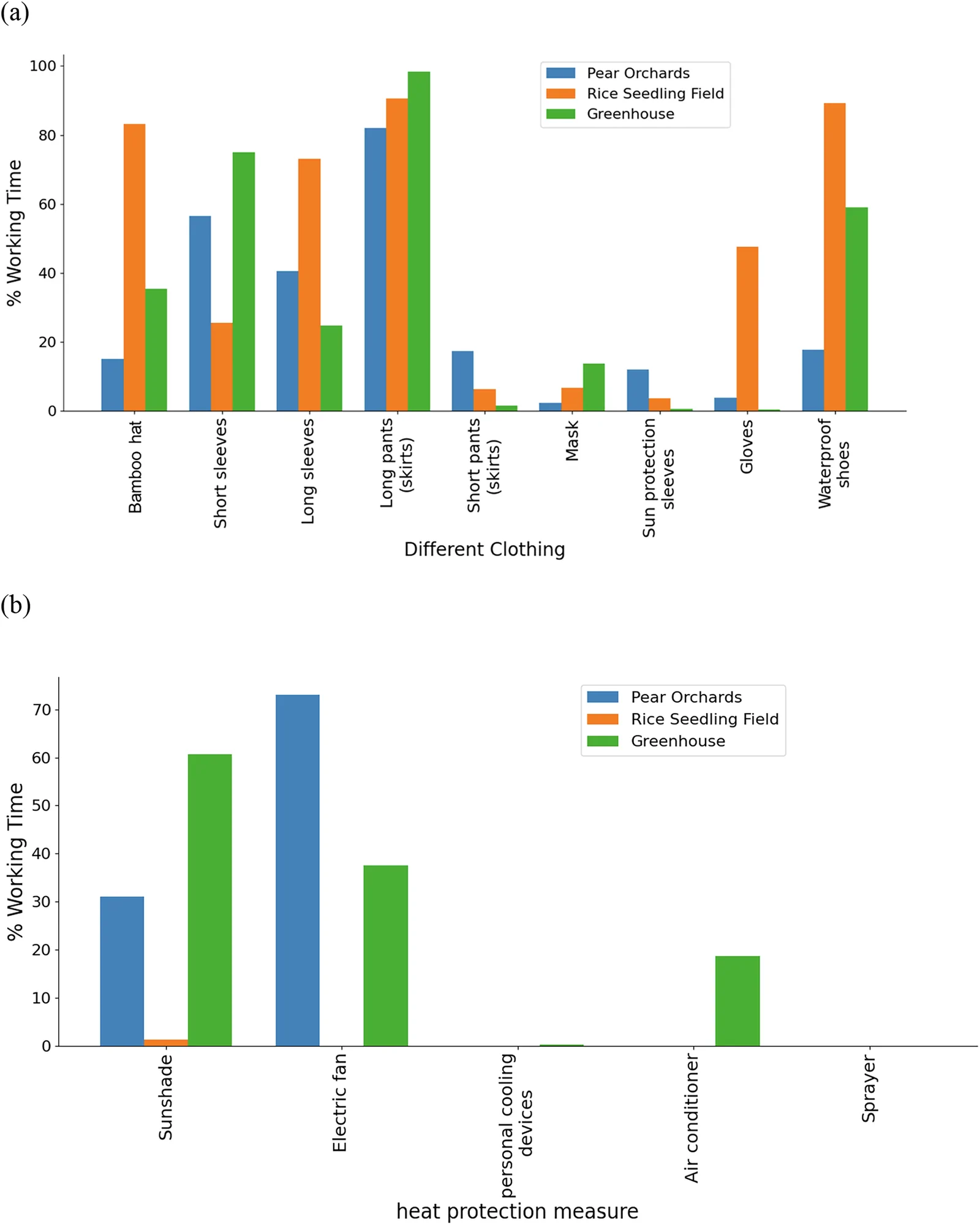
Percentage of total working time using (**a)** different clothing and (**b)** different heat protection measures among the 25 participants; 8, 6, and 11 were from the pear orchard community, rice seedling field, and greenhouse farm, respectively.

As the garments worn at work may affect heat accumulation and subsequent health impacts, the percentage of time that the participants wore different garments is summarized in Fig. 3a. Notably, the percentages of time that the participants wore a bamboo hat, short sleeves, and waterproof shoes differed among these three workplaces. Nevertheless, most participants wore long pants or skirts. Overall, the rice seedling field participants covered themselves more extensively than the other groups, with higher percentages wearing bamboo hats, long sleeves, gloves, and waterproof shoes.

Figure 3b shows heat protection measures during the monitoring periods. The pear orchards provided sunshades and electric fans, and the greenhouse farm provided sunshades, electric fans, and air-conditioners. The rice seedling field only provided sunshades for a very short period. We observed that the participants changed their conditions using these heat protection measures over a shorter time period than the 30-min intervals of the time–activity diaries. To avoid biases, these intervention factors were not evaluated further with regressions.

The garments worn at work were included in the WBGT-HRV intervention models to evaluate potential modifying effects. The results of the models excluding long pants/skirts/sleeves or short pants/skirts/sleeves are shown in Table 3b, c, respectively, with the detailed *p* values being listed in Table S6b, c. The directions (positive or negative) of the associations between the WBGT and HRV indicators when considering garments remained consistent with Table 3a. However, the percentage changes in HRV per 1 °C increase in WBGT were smaller in the two intervention models, indicating that the garments worn appear to be an effect modifier.

Wearing gloves was statistically significantly associated with an 8.7–21.2% decrease in all HRV indicators except LF/HF (Table 3b, c), and wearing masks was statistically significantly associated with an 8.8–23.3% decrease in the SDNN, LF/HF, VLF, and TP (Table 3b, c). Wearing waterproof shoes was statistically significantly associated with approximately 3% HR increases but not associated with any HRV changes. Wearing short pants/skirts and short sleeves showed inverse associations with HRV indicators (except HF) and HR compared with those of WBGT exposure, although only a few estimated coefficients reached statistical significance, possibly due to sample size limitations (Table 3b). Conversely, wearing long pants/skirts and long sleeves was associated with HRV (except HF) and HR changes in the same direction as WBGT exposure, with some estimated coefficients reaching statistical significance (Table 3c). In summary, the observed associations suggest that wearing gloves, masks, and long pants/skirts/sleeves may exacerbate HRV responses, while wearing short pants/skirts/sleeves may be protective.

## Discussion

### WBGT exposure

Previous studies have used fixed-location monitors to assess WBGT exposure for agricultural workers, including studies conducted in Thailand, India, Nigeria, Guatemala, Iran, Nicaragua, Gambia, and Brazil for rice, sugarcane, corn, plantain, coffee, vegetable, and maize farmers [10, 12, 48–55]. A summary of these publications is provided in Table S7a. In short, their mean WBGT levels ranged from 22.85 to 34.08 °C, with maximums from 32 to 36.4 °C. In comparison, this study found that pear orchard and rice seedling field workers experienced WBGT exposures of 26.7 ± 2.5 °C and 32.9 ± 4.0 °C during their outdoor working hours, respectively. These WBGT levels were within the previously reported ranges, with the exposures of the rice seedling field workers being at the higher end. Personal WBGT assessment allows for the detection of peak WBGT levels, such as 48.2 °C in the rice seedling field, which fixed-location monitors cannot capture.

Personal sensors offer a practical solution for individualized exposure assessments. In fact, the traditional fixed-location monitors may be unable to be set up where the agricultural workers are. For example, fixed-location monitors were not allowed to be located inside the greenhouse to avoid contamination of their valuable products. Small personal sensors were allowed to be carried since they were clean in front of the participants. In small-scale farming, where farmers must carry out various chores on their own, substantial interpersonal variations in WBGT exposure are to be expected. Small-scale farming is common in most Asian countries. The small and lightweight sensors used in this study are also low-cost and easy to use, providing an affordable WBGT monitoring method for these farmers for self-protection.

Efforts have been made to develop personal WBGT sensors (e.g., [21, 56]). However, these studies developed only small sensors for temperature and humidity, while solar radiation and wind speed remain difficult to measure at the personal scale. Our study advances this by incorporating a personal sensor for light intensity as a proxy for solar radiation. While light intensity sensors typically measure visible light (400–700 nm), solar radiation spans 200 nm to 4000 nm, meaning there are differences between the two. Nevertheless, given the stable relationship between light intensity and solar radiation over time (Fig. S3a, c, d), we used a site-specific linear regression for approximation. Other research groups will need to establish their own site-specific equations if adopting this approach.

Individualized WBGT monitoring helps identify high-exposure workers. For example, greenhouse participants No. 5 and 7 had the highest median WBGT exposure in greenhouse monitoring (Fig. 2). Further investigation could pinpoint specific microenvironments or tasks contributing to high heat exposure, allowing for the development of targeted adaptation strategies.

Two studies conducted in South Korea on greenhouse workers reported higher WBGT exposures inside greenhouses than outside (Table S7b) [11, 23]. In contrast, our greenhouse participants experienced lower WBGTs when working inside (25.4 ± 3.2 °C) than outside (35.5 ± 4.7 °C). While the indoor WBGT levels were within previously reported ranges, the outdoor levels were substantially higher. Taiwan’s lower latitude than South Korea, with higher temperatures and solar radiation levels during the summer, likely explains the elevated outdoor WBGT exposure observed in this study. Greenhouse farming is encouraged as a mean to reduce the impacts of extreme weathers on crops, the surprisingly high WBGT exposure and high percentages of outdoor working hours (on average 72.3%) of the greenhouse participants alarmed us to protect greenhouse workers from outdoor heat stress in subtropical areas.

Interestingly, the rice seedling field and greenhouse participants experienced higher WBGT exposure between 9:00 and 10:59 than between 14:00 and 15:59, contrary to findings in Nigeria [49] and Brazil [51]. Our previous work also observed higher WBGT levels before noon (between 10:00 and noon) than in the afternoon (noon to 14:00) in a 20-station network across Taiwan [39]. This phenomenon may be explained by Taiwan’s subtropical climate with frequent afternoon showers or thunderstorms; clouds start to form around noon, reducing solar radiation and thus lowering WBGT levels in the afternoon.

### WBGT-HRV association

Previous studies on agricultural workers’ health have focused on HR or kidney function. The two prior studies that assessed agricultural workers’ HR did not use statistical models to evaluate WBGT-HR associations [12, 48]. This study is the first to assess WBGT-HRV associations in agricultural workers. Using statistical models, it is possible to not only evaluate the heat–health association but also the net effects of intervention choices.

Previous studies have linked changes in HRV indicators to heat exposure [34, 35]. One study reported that 10 healthy males experienced significant HRV changes after a sauna at 74.3 ± 5.9 °C, with decreased RMSSD and HF, and increased LF and LF/HF. Our findings showed consistent trends in RMSSD, HF, and LF/HF, but an opposite direction in LF [34]. Another study of 296 participants in China found a negative association between hourly HRV and acute heat exposure, with a 1 °C increase (lag 1 h) leading to a 0.41% decrease in SDNN (95% CI: −0.62, −0.21) [35]. However, their participants were individuals at moderate to high cardiovascular risk, such as those with hypertension, obesity, or diabetes, limiting direct comparison with agricultural workers. Still, both studies found most HRV indicators declined with higher temperatures, consistent with our results.

PM_2.5_ is known to be associated with changes in HRV [31, 32, 57]. For instance, a 10 μg/m³ increase in PM_2.5_ was significantly linked to a 0.44–2.21% reduction in HRV indicators (except LF/HF) in 35 non-smoking participants aged 40–75 in northern Taiwan in 2018–2019 [32]. These changes are similar to those observed in this study, where a 1 °C increase in WBGT was significantly associated with a 1.05–2.44% decrease in all HRV indicators, except LF/HF. However, in this study, PM_2.5_ was not significantly associated with HRV changes, possibly due to sample size limitations or the strong association between WBGT and HRV masking the effects of PM_2.5_.

Both WBGT and PM_2.5_ exposure have been shown to impair cardiovascular autonomic functions by altering HRV, despite being driven by different mechanisms. The mechanism underlying the WBGT impacts is a thermoregulatory process where rising core temperatures necessitate blood vessel dilation, which triggers compensatory sympathetic nervous system activation to maintain blood pressure and, consequently, reduces HRV [58]. On the other hand, the mechanism of PM_2.5_ impacts is mediated by systemic inflammation and oxidative stress. When the PM_2.5_ is inhaled, it can enter the bloodstream and trigger a systemic inflammatory response, which disrupts normal autonomic control [59]. Thus, both stressors lead to the same result: an increase in sympathetic activity and a decrease in parasympathetic activity, which is reflected in a decrease in HRV.

Age and gender are well-known factors influencing heat vulnerability, with the elderly and females considered more vulnerable to heat stress [4, 47]. This study found that older participants experienced a greater decrease in certain HRV indicators, as expected. However, contrary to expectations, males showed a more significant decrease in HRV indicators in the intervention models. The small sample size of female workers (only 6) may have affected these results.

### Intervention strategies

Rest-shade-hydration is a proven strategy to reduce heat stress in the workplace [55]. For example, productivity increased in sugarcane workers in Nicaragua with regulated shaded rest periods and easy access to hydration [55]. In our study, pear-orchard workers consumed 1.5–2.8 L of water, and most rice-seedling field and greenhouse workers drank over 2.8 L during working hours. However, none had regulated hourly shaded rest periods. This strategy could be considered in Taiwan to better protect agricultural workers.

Wearing proper garments is another potential strategy to protect agricultural workers. The International Organization for Standardization (ISO) provided a clothing insulation index to evaluate the modifying effects of clothing on heat stress in 2004 [60]. However, as the garment industry has introduced various materials over the years, the original ISO procedures may not fully capture the current insulation possibilities. Moreover, certain clothing choices, such as wearing long sleeves and bamboo hats, may offer protection against solar radiation but hinder sweat evaporation, complicating assessments of their net impacts on human physiological functions. This study used a combination of personal monitoring, time–activity diaries, and statistical models to evaluate the modifying effects of garments worn at work. It showed that the net effects of wearing gloves, masks, and long sleeves/pants/skirts are actually exacerbating HRV responses. On the other hand, the protective effect of wearing a bamboo hat may counterbalance these negative effects; no statistically significant association was found for either increased or decreased HRV changes. Wearing short sleeves/pants/skirts may be protective, but sunscreen needs to be applied to prevent skin burn.

### Innovation, implications, and limitations

Under climate change, agricultural workers are among the most heat-exposed groups. This study addresses research gaps for agricultural workers, presenting two innovations: Firstly, personal sensors were applied to assess WBGT exposure, providing proximate measurements for individual workers performing different tasks at varying locations. Substantial interpersonal variations in heat exposure were revealed, with the ambient WBGT levels underestimating the mean exposure, maximum exposure, and percentages above the critical thresholds of 32.2 °C and 36 °C for outdoor participants who were not in the shade. This highlights the need for personal WBGT exposure assessments. Furthermore, OSHA has accepted the use of Liljegren equations since 2017 for estimating WBGT levels, and several studies have applied the equations at the city or regional scales [18, 19, 61, 62]. This pioneering study uniquely combined these equations with personal sensors to estimate personal WBGT levels in actual field conditions.

Secondly, this study assessed the WBGT-HRV association using medically certified biosensors that were worn by individual participants over two 48-h sessions. Statistically significant associations were observed between WBGT exposure and immediate changes in HRV indicators. Potential intervention choices, such as garments, were also evaluated. This methodology can be adapted for other outdoor workers to evaluate the impacts of heat and different adaptation methods.

This panel study aims to take advantage of the detailed records of individual agricultural workers to assess their WBGT exposure, its association with HRV indicators, and intervention factors. It is not intended to generalize this WBGT-HRV relationship to other populations. Nevertheless, the assessed WBGT levels were good indications of the WBGT ranges that can be experienced under similar environmental conditions, and the evaluated intervention choices serve as a good reference for other populations.

The three studied workplaces were chosen to assess differences in working conditions and heat-reduction measures due to the scale of their business, i.e., the company-owned greenhouse farm versus small-scale farming (the pear orchards and rice seedling field). The survey results indicated lower water consumption in self-employed pear orchard participants than those in the other two workplaces. Differences in heat protection measures were also observed, with the greenhouse farm providing more equipment than the other two workplaces. No conclusion can be reached based on only three workplaces; more studies must be conducted to assess the influence of business scale on heat adaptation strategies. Nevertheless, even with shades, fans, and sufficient drinking water, statistically significant HRV changes were still observed for these agricultural workers. This shows the need for additional effective adaptation strategies to reduce workers’ heat-related health risks.

The high percentages of WBGTs above 32.2 and 36 °C and statistically significant associations with HRV reduction highlight the urgent need for heat health adaptation strategies to protect agricultural workers in Taiwan. Encouraging workers to wear short sleeves/pants/skirts and apply sunscreen may be a potential tactic to reduce the impacts of heat stress on cardiovascular systems. Currently, the Ministry of Labor in Taiwan regards small-scale farmers (who account for the majority of agricultural workers) as being under the jurisdiction of the Ministry of Agriculture, which primarily focuses on crop protection under climate change. This study provides scientific evidence to support the need for specific heat adaptation strategies targeting agricultural workers to limit health risks.

Besides the intervention factors that were evaluated in this study, another recommended adaptation strategy involves implementing a WBGT warning system, allowing outdoor workers to plan ahead and minimize heat-related health risks. The OSHA, USA, has proposed heat safety rules using the WBGT and Heat Index (HI) as exposure indicators. The HI is used for screening, since it fails to account for solar radiation and wind speed, which are key factors for outdoor heat stress assessments. However, the HI, unlike the WBGT, is forecasted by the National Weather Service, USA. Traditionally, weather agencies worldwide routinely monitor and forecast the temperature, but not the natural wet-bulb temperature and globe temperature, hindering their progress in providing WBGT monitoring and forecast services. Nevertheless, the Liljegren equations use standard meteorological data to calculate the WBGT, overcoming these limitations. Taiwan’s Central Weather Administration has forecasted the WBGT since 2021; this open service is valuable for resource-limited small-scale farmers. However, the collaboration with the Ministries of Labor and Agriculture must be strengthened to reach individual farmers. In addition, a European occupational warning system based on the HEAT-SHIELD project was established using the Liljegren equations [61, 62]; nevertheless, it is not clear whether this system is for research purposes only or can be taken up by occupational agencies. To align with the “early warning for all” principle emphasized by the WMO (https://wmo.int/activities/early-warnings-all/wmo-and-early-warnings-all-initiative), for countries without heat warning systems, we suggest establishing a WBGT warning system, which could be achieved using Liljegren equations with the routinely forecasted meteorological parameters from weather agencies as inputs. Both employers and employees in the workplace can plan ahead according to the forecast to reduce heat exposure.

There are several limitations of this study. First, the regression of light intensity and solar radiation was suitable for Taiwan; researchers in other countries will need to develop their own equations. Additionally, time–activity diaries at a 30-min resolution may be crude in combination with 1-min sensor data. However, it is difficult to ask participants to record something in intervals shorter than 30 min; other smart technologies may need to be developed to overcome this drawback. Moreover, shade and the body orientation of participants may influence the accuracy of light intensity measurements. Nevertheless, since the participants were constantly moving, such an interference may not be very substantial. In addition, while the models adjusted for several environmental confounders, residual confounding—particularly from ozone—remains possible. Sensitivity analyses using ambient ozone data from nearby monitoring stations showed minimal impact on WBGT–HRV associations, supporting the robustness of our findings. Furthermore, some coefficients estimating the modifying effects of garments did not reach statistical significance, possibly due to the limited sample size. Lastly, due to the limited sample size, the associations of HRV-WBGT and the intervention effects of garments should be interpreted with caution. It is not intended to generalize these WBGT-HRV relationships to other populations. Nevertheless, this study successfully demonstrated the use of epidemiological models to evaluate the modifying effects of garments in the field.

In conclusion, this study introduces an innovative methodology combining small and lightweight personal sensors with OSHA-approved Liljegren equations for WBGT assessments and for evaluating WBGT-HRV associations. Our fieldwork in three agricultural workplaces in Taiwan revealed substantial interpersonal WBGT variations and demonstrated the advantage of personal sensors for capturing peak heat exposures. The highest percentage of individual WBGT exposure exceeding 36 °C was 44.2%; this demonstrated a high level of heat stress exposure. The ambient WBGT levels from the fixed-location monitors underestimated the mean exposure (1.9 °C for the rice seedling participants and 3.2 °C for the greenhouse participants), maximum exposure (4.2 °C for the rice seedling participants and 7 °C for the greenhouse participants), and percentages above the critical thresholds of 32.2 and 36 °C for outdoor participants who were not in the shade. Moreover, the rice seedling field and greenhouse participants experienced higher WBGT exposure levels between 9:00 and 10:59 than between 14:00 and 15:59 due to subtropical weather patterns, contrary to findings in other countries. This further emphasizes the importance of local in situ monitoring. The small, lightweight, low-cost, and easy-to-use sensors that were used in this study provide an affordable WBGT monitoring method for farmers for self-protection.

Regarding WBGT-HRV relationships, a 1 °C WBGT increase was associated with statistically significant 1.05–2.44% decreases in HRV indicators, except for LF/HF, and a 0.65% increase in HR. Our intervention models revealed that wearing gloves, masks, and long garments may exacerbate heat stress, while wearing short garments may be protective. The protective effects of wearing a bamboo hat may counterbalance the negative effects. The presented exposure measurements and epidemiological technology/methodology offer excellent potential for researchers across the world to assess personal WBGT levels, their association with health impacts, and adaptation choices for people who work or exercise outdoors, ultimately contributing to formulating heat adaptation strategies for health risk reduction in the face of climate change.

## Supplementary information

Supplementary information

STROBE Statement—Checklist

## Data Availability

Academia Sinica, RCEC. (2025). Heat exposure of agricultural workers (Version 2025-01-20T00:42:39.968238) [dataset]. https://pid.depositar.io/ark:37281/k5s9f6459; time–activity diaries and HRV data are not provided due to privacy concerns.
