# Supplementary material for: An innovative method of evaluating the association of heat exposure and heart rate variability in a panel of agricultural workers with small and lightweight personal sensors
Source: J Expo Sci Environ Epidemiol. 2026 Mar 17;36(6):1060–77. doi: 10.1038/s41370-026-00848-9 (PMC13727664; doi:10.1038/s41370-026-00848-9)
Supplement: Supplementary file 1 [file 41370_2026_848_MOESM1_ESM.docx]

Supplementary Information

An innovative method of evaluating the association of heat exposure and heart rate variability in a panel of agricultural workers with small and lightweight personal sensors

Shih-Chun Candice Lung^1,2,3^*, Shu-Chuan Hu^1^, Cheng-You Tsai^1^, Ming-Chien Mark Tsou^1^, Jou-Chen Joy Yeh^1^, Chun Hu Liu^1^

^1^Research Center for Environmental Changes, Academia Sinica, Nangang, Taipei, Taiwan 115.

^2^Department of Atmospheric Sciences, National Taiwan University, Taipei, Taiwan.

^3^Institute of Environmental and Occupational Health Sciences, National Taiwan University, Taipei, Taiwan.

^*^Corresponding author, Research Center for Environmental Changes, Academia Sinica, No. 128, Sec. 2, Academia Rd, Nangang, Taipei, Taiwan 11529; Tel: 886-2-27875908, Fax: 886-2-27833584, email: *sclung@rcec.sinica.edu.tw.*

**Summary:**

Text: WBGT equations and Liljegren equations (pages 3-5)

Table S1: Skewness and Kurtosis of the log transformation; (page 6)

S2: Model selection; (pages 7-8)

S3: Environmental/personal levels; (pages 9-11)

S4: Working hours; (page 12)

S5: WBGT above 32.2 ℃ and 36 ℃; (page 13)

S6: WBGT-heart-rate-variability associations (*p*-values); (pages 14-17)

S7: WBGT-monitoring publications; (pages 18-21)

Figure S1: Temperature and humidity sensor comparisons; (page 22)

S2: Photo of a participant wearing sensors; (Page 23)

S3: Light-intensity and solar-radiation regressions; (pages 24-25)

S4: Nonlinearity between certain dependent and independent variables; (page 26)

S5: PM_2.5_ distributions; (page 27)

S6: Summary of the interview results; (pages 28-30)

S7: WBGT during working/non-working hours; (pages 31-32)

S8-S11: Diurnal temperature, relative-humidity, solar-radiation, and wind-speed, respectively; (pages 33-36)

S12: At-work WBGT in different classifications; (page 37)

S13: Participants’ diurnal activity-levels. (page 38)

**WBGT equations (OSHA, 2017):**

WBGTout = 0.7Tw + 0.2Tg + 0.1Ta (outdoors)	Eq. (1)

WBGTin = 0.7Tw + 0.3Tg (indoors)	Eq. (2)

Ta: dry temperature, Tw: natural wet-bulb temperature, and Tg: globe temperature

**Liljegren’s equations: (Liljegren et al., 2008)**

For natural wet-bulb temperature (Tw)

$Tw=Ta-\frac{k_{x}\Delta HM_{H2O}}{h}\left( \frac{e_{w}-e_{a}}{P-e_{w}} \right)+\frac{\Delta F_{net}}{Ah}$	Eq.(3)

$$\Delta F_{net}=\frac{A}{2}{\sigmaɛ}_{w}\left( ɛ_{a}T_{a}^{4}+ɛ_{sfc}T_{sfc}^{4} \right)-{A\sigmaɛ}_{w}T_{a}^{4}+A_{1}\left( 1-\alpha_{w} \right)\left( 1-f_{dir} \right)S$$

$+A_{2}\left( 1-\alpha_{w} \right)f_{dir}S/\cos\left( \theta\right)+A\left( 1-\alpha_{w} \right)\alpha_{sfc}S$	Eq.(4)

$A=\pi DL$	Eq.(5)

$A_{1}=\pi DL+\frac{\pi D^{2}}{4}=A\left( 1+D/4L \right)$	Eq.(6)

$A_{2}=\pi DL\times sin \left( \theta\right)+\pi D^{2}/4\times\cos\left( \theta\right)=A\left( \sin\left( \theta\right)/\pi+\cos\left( \theta\right)\times D/4L \right)$	Eq.(7)

*A:* the surface area of wet blub sensor wick(m^2^)

$\Delta H$: the heat of vaporization (W/kg)

$ɛ_{w}$: emissivity of the wick, 0.95

$ɛ_{a}$: emissivity of atmosphere, $ɛ_{a}=0.575e_{a}^{0.143}$

$e_{a}$: the partial pressure of water vapor, $e_{a}=RHe_{w}(T_{a})$ hPa

$e_{w}$ the saturation vapor pressure at temperature $T_{a}$ and air pressure P, $e_{w}\left( T_{a}, P \right)= (1.0007+(3.46\times{10}^{-6}\times P))\times(6.1121\times e^{17.502\times(T_{Air}-273.15)/(T_{Air}-32.18)})$ hPa

$ɛ_{sfc}$: emissivity of the surface ($ɛ_{sfc}T_{sfc}^{4}\approx T_{a}^{4}$)

*h*: the convective heat transfer coefficient, $h=0.281\frac{k}{D} {Re}^{0.6}{Pr}^{0.44}$ (W/(m^2^-K)) ; k is he convective mass transfer coefficient; *Re* is Reynold number; *Pr* is Rrandtl number; *D* is the diameter of the wick

$\Pr= \frac{C_{p}}{C_{p}+1.25\frac{R_{gas}}{M_{Air}}}$ (polyatomic gas)

$k =(Cp+1.25\frac{R_{gas}}{M_{Air}})\mu$, thermal conductivity

$Re =\frac{\rho VD}{\mu}$

$C_{p}$: specific heat capacity, 1003.5 J/kg-K

$R_{gas}$: universal gas constant, 8314.34 J/kg mol-K

$\mu$: viscosity of air, kg/(m-s)

V: wind speed, m/s

*P*: the barometric pressure (hPa)

$M_{H2O}$: the molecular weight of water vapor, 18.015 g/mole

$M_{Air}$: the molecular weight of air, 28.97 g/mole

$\Delta F_{net}$: the net radiant heat flux (W)

$\alpha_{w}$: the albedo of wick, 0.4

$\alpha_{sfc}$: the surface albedo

$\theta$: the solar zenith angle

$f_{dir}$: the ratio of the direct horizontal solar irradiance to the total horizontal solar irradiance

$T_{a}$: air temperature

$T_{w}$: wet bulb temperature

For global temperature (Tg):

$$Aɛ_{g}\sigma T_{g}^{4}+Ah\left( T_{g}-T_{a} \right)=\frac{A}{2}ɛ_{g}\sigma\left( ɛ_{a}T_{a}^{4}+ɛ_{sfc}T_{sfc}^{4} \right)$$

$$+\frac{A}{2}\left( 1-\alpha_{g} \right)\left( 1-f_{dir} \right)S$$

$$+\frac{\frac{A}{4}\left( 1-\alpha_{g} \right)f_{dir}S}{\cos\left( \theta\right)}$$

$+\frac{A}{2}\left( 1-\alpha_{g} \right)\alpha_{sfc}S$	Eq.(8)

*A*: the surface area of black ball (m^2^)

$ɛ_{g}$: the globe emissivity, 0.95

$ɛ_{a}$: emissivity of atmosphere, $ɛ_{a}=0.575e_{a}^{0.143}$

$e_{a}$: the partial pressure of water vapor, $e_{a}=RHe_{w}(T_{a})$ hPa

$e_{w}$: the saturation vapor pressure at temperature $T_{a}$ and air pressure P, $e_{w}\left( T_{a}, P \right)= (1.0007+(3.46\times{10}^{-6}\times P))\times(6.1121\times e^{17.502\times(T_{Air}-273.15)/(T_{Air}-32.18)})$ hPa

$ɛ_{sfc}$: emissivity of the surface ($ɛ_{sfc}T_{sfc}^{4}\approx T_{a}^{4}$)

*h*: the convective heat transfer coefficient (W/(m^2^-K)); $h=(\frac{k}{D})Nu$；$Nu=2.0+0.6{Re}^{1/2}{Pr}^{1/3}$; *Nu* is Nusselt number; *Re* is Reynold number; *Pr* is Rrandtl number; *D* is the diameter of the black ball

$\Pr= \frac{C_{p}}{C_{p}+1.25\frac{R_{gas}}{M_{Air}}}$ (polyatomic gas)

$k =(Cp+1.25\frac{R_{gas}}{M_{Air}})\mu$, thermal conductivity

$Re =\frac{\rho VD}{\mu}$

$C_{p}$: specific heat capacity, 1003.5 J/kg-K

$R_{gas}$: universal gas constant, 8314.34 J/kg mol-K

$\mu$: viscosity of air, kg/(m-s)

V: wind speed, m/s

*P*: the barometric pressure (hPa)

$M_{H2O}$: the molecular weight of water vapor, 18.015 g/mole

$M_{Air}$: the molecular weight of air, 28.97 g/mole

$\sigma$: the Stefan-Boltzmann constant, $5.6696\times{10}^{-8}$ W/(m^2^-K^4^)

$T_{sfc}$: the temperature of the surface (K)

*S*: the solar irradiance (W/m^2^)

$\alpha_{g}$: the albedo of ground, 0.05

$\alpha_{sfc}$: the surface albedo

$\theta$: the solar zenith angle

$f_{dir}$: the ratio of the direct horizontal solar irradiance to the total horizontal solar irradiance

$T_{a}$: air temperature

$T_{g}$: globe temperature

After obtaining Tw and Tg based on Liljegren’s equations, WBGT in the outdoor and indoor environments can be calculated.

Reference:

Occupational Safety and Health Administration (OSHA). 2017. Chapter 4 Heat Stress, OSHA Technical Manual (OTM) Section III, updated: September 15, 2017. https://www.osha.gov/otm/section-3-health-hazards/chapter-4#introduction.

Liljegren JC, Carhart RA, Lawday P, Tschopp S, Sharp R. Modeling the Wet Bulb Globe Temperature using standard meteorological measurements. J Occup Environ Hyg. 2008;5(10):645-55. https://doi.org/10.1080/15459620802310770.

Table S1 Skewness and kurtosis values before and after log10 transformation for HRV indices.

|  | Skewness |  | Kurtosis |  |
| --- | --- | --- | --- | --- |
| HRV indices | Before transformation | After transformation | Before transformation | After transformation |
| SDNN | 2.022 | 0.144 | 5.735 | -0.042 |
| RMSSD | 1.080 | -0.367 | 2.553 | -0.489 |
| LF/HF ratio | 1.936 | -0.571 | 5.431 | 0.565 |
| HF | 4.562 | 0.072 | 29.428 | -0.413 |
| LF | 5.739 | 0.303 | 47.179 | 0.137 |
| VLF | 5.380 | 0.184 | 40.108 | -0.034 |
| TP | 5.094 | 0.261 | 35.201 | 0.006 |

Table S2(a) Akaike Information Criterion (AIC)-based model comparison across varying fixed and random effect structures in GAMM.

| Model | AIC |
| --- | --- |
| Model 1: Y= PM2.5 + Age_G + BMI_G + Activitymean + Gender + WBGT +Time, random = list(SubjID = ~1), | -9155.075 |
| Model 2: Y= PM2.5 + Age_G + BMI_G + **s(Activitymean,bs=c("tp"))** + Gender + WBGT + Time, random = list(SubjID = ~1), | -9171.484 |
| Model 3: Y= PM2.5 + Age_G + BMI_G + Activitymean + Gender + WBGT + **s(Time,bs=c("cc"))**, random = list(SubjID = ~1), | -9192.150 |
| Model 4: Y= PM2.5 + Age_G + BMI_G + **s(Activitymean,bs=c("tp"))** + Gender + WBGT + **s(Time,bs=c("cc"))**, random = list(SubjID = ~1), | -9207.447  (Best and selcetion) |
| Model 5: Y= PM2.5 + Age_G + BMI_G + s(Activitymean,bs=c("tp")) + Gender + WBGT + s(Time,bs=c("cc")), random = list(**FarmID = ~1**, SubjID = ~1), | -9205.447 |

Model 1: A linear mixed-effects model including all predictors as linear terms. A random intercept for each participant (SubjID) was included to account for within-participant correlation.

Model 2: Extended Model 1 by modeling activity intensity (Activitymean) as a smooth term using a thin plate spline (basis = "tp") to capture potential nonlinear effects of physical activity. A random intercept for each participant (SubjID) was included.

Model 3: Similar to Model 1, but Time was modeled as a cyclic cubic spline (basis = "cc") to capture possible periodic nonlinear time effects. A random intercept for each participant (SubjID) was included.

Model 4: Included smooth terms for both activity intensity (Activitymean) and Time, allowing for simultaneous nonlinear relationships with the outcome. A random intercept for each participant (SubjID) was included. **Model 4 was selected as the final model due to the lowest AIC.**

Model 5: Same as Model 4 but incorporated two levels of random effects: a random intercept for each participant (SubjID) and a nested random intercept for the work environment (FarmID).

Table S2(b) Main model and two intervention models

Model

**Main Model:** Y= PM2.5 + Age_G + BMI_G + **s(Activitymean,bs=c("tp"))** + Gender + WBGT + **s(Time,bs=c("cc"))**, random = list(SubjID = ~1),

**Intervention Model 1**: Y= PM2.5 + Age_G + BMI_G + **s(Activitymean,bs=c("tp"))** + Gender + WBGT + **s(Time,bs=c("cc"))** + bamboo hats + short sleeves + short pants/skirts +masks +gloves +waterproof shoes, random = list(SubjID = ~1),

**Intervention Model 2:** Y= PM2.5 + Age_G + BMI_G + **s(Activitymean,bs=c("tp"))** + Gender + WBGT + **s(Time,bs=c("cc"))** + bamboo hats + long sleeves + long pants/skirts +masks +gloves +waterproof shoes, random = list(SubjID = ~1),

Note: activity intensity (Activitymean) was modeled as a smooth term using a thin plate spline (basis = "tp") to capture potential nonlinear effects of physical activity; Time was modeled as a cyclic cubic spline (basis = "cc") to capture possible periodic nonlinear time effects; a random intercept for each participant (SubjID) was included; garment terms were all dummy variables.

Table S3 (a) Temperature, relative humidity, solar radiation, and wind speed in a 5-min resolution during the entire monitoring period (non-sleeping periods plus sleeping periods excluding rainy periods); observations of temperature, relative humidity, and solar radiation were obtained from personal sensors while those of wind speed were from the on-site weather station

|  |  | **Pear Orchards** | **Rice seedling Field** | **Greenhouse** | **Overall**  **(25 participants)** |
| --- | --- | --- | --- | --- | --- |
|  |  | **(8 participants)** | **(6 participants)** | **(11 participants)** |  |
| **Temperature (℃)** | Mean±SD | 29.1±2.3 | 32.1±4.2 | 29.7±3.8 | 30.0±3.7 |
|  | Max | 41.4 | 56.2 | 45.0 | 56.2 |
|  | Sample size | 12517 | 8778 | 17609 | 38904 |
| **Relative Humidity (%)** | Mean±SD | 73.6±8.5 | 65.0±9.7 | 61.6±10.3 | 66.2±10.9 |
|  | Max | 98.0 | 99.4 | 95.8 | 99.4 |
|  | Sample size | 12517 | 8778 | 17609 | 38904 |
| **Solar Radiation (w/m^2^)** | Mean±SD | 6.5±24.5 | 84.2±156.6 | 133.5±279.5 | 81.5±210.0 |
|  | Max | 564.3 | 1000.0 | 1000.0 | 1000.0 |
|  | Sample size | 12517 | 8778 | 17609 | 38904 |
| **Wind Speed (m/s)** | Mean±SD | 0.3±0.2 | 0.8±0.8 | 0.3±0.2 | 0.4±0.5 |
|  | Max | 2.2 | 4.7 | 2.1 | 4.7 |
|  | Sample size | 12517 | 8778 | 17609 | 38904 |

Table S3 (b) Ambient temperature, relative humidity, solar radiation, and wind speed in a 5-min resolution from the on-site weather stations during the entire monitoring period (excluding rainy periods); these observations were obtained in the ambient air and not in the microenvironments of the participants stayed

|  |  | **Pear Orchards** | **Rice seedling Field** | **Greenhouse** | **Overall** |
| --- | --- | --- | --- | --- | --- |
| **Temperature (℃)** | Mean ± SD | 29.9 ± 2.3 | 31.8 ± 1.3 | 31.8 ± 2.1 | 31.2 ± 1.9 |
|  | Max | 33.4 | 33.9 | 35.6 | 35.6 |
|  | Sample size | 859 | 1061 | 880 | 2800 |
| **Relative Humidity (%)** | Mean ± SD | 72.1 ± 9.8 | 71.6 ± 5.5 | 67.7 ± 9.0 | 70.5 ± 8.1 |
|  | Max | 96.8 | 93.3 | 93.4 | 96.8 |
|  | Sample size | 859 | 1061 | 880 | 2800 |
| **Solar Radiation (w/m^2^)** | Mean ± SD | 410.5 ± 282.7 | 502.1 ± 299.9 | 515.1 ± 236.9 | 475.9 ± 273.2 |
|  | Max | 1057.8 | 1020.4 | 1068 | 1068.0 |
|  | Sample size | 859 | 1061 | 880 | 2800 |
| **Wind Speed (m/s)** | Mean ± SD | 0.5 ± 0.5 | 1.8 ± 1.0 | 0.6 ± 0.5 | 1.0 ± 0.7 |
|  | Max | 2.2 | 4.7 | 2.1 | 4.7 |
|  | Sample size | 859 | 1061 | 880 | 2800 |

Table S3(c) PM_2.5_ in a 5-min resolution during the non-sleeping period (excluding rainy periods), observations were obtained from the on-site indoor or outdoor sensors which was the closest to the participants in the microenvironments where the participants stayed

|  |  | **Pear Orchards** | **Rice seedling Field** | **Greenhouse** | **Overall**  **(25 participants)** |
| --- | --- | --- | --- | --- | --- |
|  |  | **(8 participants)** | **(6 participants)** | **(11 participants)** |  |
| **Personal** | Mean±SD | 10.8±2.9 | 11.3±4.8 | 12.5±3.6 | 11.8±3.8 |
| **PM_2.5_ (µg/m^3^)** | Max | 28.2 | 41.9 | 41.8 | 41.9 |
|  | Sample size | 6761 | 5666 | 12359 | 24786 |

Table S4 The mean, standard deviation (SD), and maximum hours of the participants spent at different micro-environments; the percentage is the percentage of mean hours spent at that micro-environment during the working or non-working hours

|  | Working hours (hr) | | | Non-working hours (hr) | | | | | |
| --- | --- | --- | --- | --- | --- | --- | --- | --- | --- |
|  | Indoor | Outdoor | Overall Mean | Sleep | Indoor | Outdoor | Transportation | Transportation | Overall Mean |
|  |  |  |  |  |  |  | (indoor) | (outdoor) |  |
| **Pear Orchards (8 participants)** | | | |  |  |  |  |  |  |
| Mean±SD | 6.7±2.8 | 2.8±2.6 | 9.5 | 7.0±0.7 | 3.3±1.3 | 0.1±0.1 | 0.8±0.6 | 0.8±0.8 | 12.0 |
| % | 70.3% | 29.7% |  | 59.1% | 27.2% | 0.7% | 6.3% | 6.7% |  |
| max | 10.8 | 8.8 |  | 7.9 | 5.3 | 0.2 | 1.8 | 2.1 |  |
| **Rice Seedling Field (6 participants)** | | | |  |  |  |  |  |  |
| Mean±SD | 2.4±1.1 | 6.4±0.9 | 8.8 | 7.1±0.7 | 4±1.2 | 1±0.7 | 0.3±0.1 | 0.5±0.2 | 12.9 |
| % | 28.9% | 71.1% |  | 56.7% | 30.7% | 7.0% | 1.6% | 4.0% |  |
| max | 4.1 | 12.3 |  | 7.8 | 5.5 | 1.8 | 0.3 | 0.8 |  |
| **Greenhouse (11 participants)** | | | |  |  |  |  |  |  |
| Mean±SD | 2.1±0.7 | 5.4±0.8 | 7.5 | 7.6±0.9 | 4±1.2 | 0.6±0.2 | 0.9±1 | 1.2±0.3 | 14.3 |
| % | 27.7% | 72.3% |  | 54.8% | 28.8% | 4.6% | 3.3% | 8.4% |  |
| max | 3.6 | 6.5 |  | 8.7 | 5.8 | 0.9 | 2.8 | 1.7 |  |
| **Overall Mean** | 3.6±2.7 | 4.8±2.1 |  | 7.3±0.8 | 3.8±1.2 | 0.5±0.5 | 0.7±0.7 | 0.9±0.6 |  |

Table S5 Percentages of WBGT exposure higher than 32.2 ℃ and 36 ℃ for each participant in the (a) pear orchards, (b) rice seedling field, and (c) greenhouse farm as well as those in (d) different gender, age, and body mass index (BMI) categories; P denotes each participant

| **(a)** | **P1** | **P2** | **P3** | **P4** | **P5** | **P6** | **P7** | **P8** | **Ambient** |  |  |  |
| --- | --- | --- | --- | --- | --- | --- | --- | --- | --- | --- | --- | --- |
| **% ≥ 32.2℃** | 0 | 0 | 0 | 0.42 | 0 | 1.62 | 0.69 | 1.17 | 22.6 |  |  |  |
| **% ≥ 36℃** | 0 | 0 | 0 | 0 | 0 | 0 | 0 | 0 | 2.6 |  |  |  |
|  |  |  |  |  |  |  |  |  |  |  |  |  |
| **(b)** | **P1** | **P2** | **P3** | **P4** | **P5** | **P6** | **Ambient** |  |  |  |  |  |
| **% ≥ 32.2℃** | 31.9 | 27.2 | 40.8 | 42.9 | 60.8 | 57.7 | 34.9 |  |  |  |  |  |
| **% ≥ 36℃** | 1.7 | 15.6 | 19.1 | 18.6 | 23.8 | 26.0 | 0.3 |  |  |  |  |  |
|  |  |  |  |  |  |  |  |  |  |  |  |  |
| **(c)** | **P1** | **P2** | **P3** | **P4** | **P5** | **P6** | **P7** | **P8** | **P9** | **P10** | **P11** | **Ambient** |
| **% ≥ 32.2℃** | 27.3 | 42.8 | 56.5 | 58.9 | 62.2 | 55.4 | 61.2 | 61.5 | 52.1 | 66.3 | 65.7 | 45.8 |
| **% ≥ 36℃** | 12.2 | 31.9 | 35.2 | 36.8 | 44.2 | 30.8 | 41.8 | 40.1 | 30.8 | 41.8 | 37.1 | 11.1 |
|  |  |  |  |  |  |  |  |  |  |  |  |  |
| **(d)** | **Male** | **Female** | **Age<40** | **Age ≥40** | **BMI<24** | **BMI≥ 24** |  |  |  |  |  |  |
| **% ≥ 32.2℃** | 37.6 | 14.7 | 49.9 | 10.1 | 29.7 | 33.0 |  |  |  |  |  |  |
| **% ≥ 36℃** | 20.3 | 8.4 | 29.6 | 2.8 | 16.4 | 17.6 |  |  |  |  |  |  |

Table S6 The association of WBGT and heart rate variability (HRV) indicators in a 5-min resolution in (a) non-sleeping periods (excluding raining periods) (n=24786), (b) working hours considering clothing conditions excluding long sleeves and long pants (n=9752), and (c) working hours considering clothing conditions excluding short sleeves and short pants (n=9752). The numbers in the table are percent changes of the respective HRV indicators for 1 ℃ of WBGT increase or 1 μg/m^3^ of PM_2.5_ increase. The detailed p-values were listed. Note: *: 0.01 ≦ *p* < 0.5; **: 0.001 ≦ *p <* 0.01; ***: *p* < 0.001

| (a) | SDNN |  | RMSSD |  | LF/HF |  | LF |  | HF |  |
| --- | --- | --- | --- | --- | --- | --- | --- | --- | --- | --- |
|  | % change | Pr(>\|t\|) | % change | Pr(>\|t\|) | % change | Pr(>\|t\|) | % change | Pr(>\|t\|) | % change | Pr(>\|t\|) |
| WBGT | -1.13*** | <0.001*** | -1.05*** | <0.001*** | 0.625*** | <0.001*** | -2.14*** | <0.001*** | -2.44*** | <0.001*** |
| Age ≥ 40 | -22.3* | 0.0424* | -33.3** | 0.00702** | 5.92 | 0.654 | -52.8** | 0.00642** | -55.4* | 0.0114* |
| Male | -2.80 | 0.840 | -19.3 | 0.207 | 59.5** | 0.00134** | -23.1 | 0.400 | -51.9* | 0.0434* |
| Overweight/obese | -10.8 | 0.289 | -9.01 | 0.470 | -13.4 | 0.198 | -26.8 | 0.193 | -15.6 | 0.541 |
| PM_2.5_ | -0.141 | 0.181 | -0.185 | 0.125 | 0.340* | 0.041* | -0.138 | 0.564 | -0.480 | 0.0871 |
|  |  |  |  |  |  |  |  |  |  |  |
| (a) continued | VLF |  | TP |  | HR |  |  |  |  |  |
|  | % change | Pr(>\|t\|) | % change | Pr(>\|t\|) | % change | Pr(>\|t\|) |  |  |  |  |
| WBGT | -1.43*** | <0.001*** | -1.69*** | <0.001*** | 0.647*** | <0.001*** |  |  |  |  |
| Age ≥ 40 | -35.6 | 0.0549 | -42.5* | 0.0215* | -2.95 | 0.399 |  |  |  |  |
| Male | 7.91 | 0.770 | -12.8 | 0.616 | -1.44 | 0.719 |  |  |  |  |
| Overweight/obese | -20.7 | 0.247 | -20.5 | 0.274 | 1.34 | 0.666 |  |  |  |  |
| PM_2.5_ | -0.309 | 0.150 | -0.290 | 0.160 | 0.0374 | 0.158 |  |  |  |  |
|  |  |  |  |  |  |  |  |  |  |  |
| (b) | SDNN |  | RMSSD |  | LF/HF |  | LF |  | HF |  |
|  | % change | Pr(>\|t\|) | % change | Pr(>\|t\|) | % change | Pr(>\|t\|) | % change | Pr(>\|t\|) | % change | Pr(>\|t\|) |
| WBGT | -0.742*** | <0.001*** | -0.849*** | <0.001*** | -0.413 | 0.062 | -1.73*** | <0.001*** | -1.40*** | <0.001*** |
| Short sleeves | 3.49 | 0.0569 | 2.69 | 0.288 | 3.22 | 0.235 | 14.0** | 0.00443** | 9.94 | 0.0959 |
| Short pants (skirts) | 7.12 | 0.0811 | 2.68 | 0.628 | 12.0 | 0.0507 | 1.74 | 0.864 | -8.55 | 0.473 |
| Gloves | -8.68*** | <0.001*** | -10.8** | 0.00272** | 3.52 | 0.398 | -18.5** | 0.00353** | -21.2** | 0.00620** |
| Mask | -9.12*** | <0.001*** | 1.70 | 0.648 | -11.4** | 0.00323** | -4.87 | 0.470 | 6.04 | 0.495 |
| Waterproof shoes | -0.19 | 0.932 | -2.33 | 0.418 | -5.62 | 0.0935 | -6.31 | 0.239 | 1.19 | 0.865 |
| Bamboo hat | 1.73 | 0.373 | -0.859 | 0.736 | -1.66 | 0.562 | -1.90 | 0.689 | -0.899 | 0.880 |
| Age ≥ 40 | -22.1* | 0.0199* | -31.8* | 0.0165* | -9.54 | 0.524 | -56.8** | 0.00216** | -52.2* | 0.0369* |
| Male | -15.21 | 0.175 | -36.8 | 0.011273 | 75.6** | 0.00158** | -50.4* | 0.0238* | -71.5** | 0.00174** |
| Overweight/obese | -3.63 | 0.692 | -0.535 | 0.969 | -15.7 | 0.209 | -16.1 | 0.460 | -1.19 | 0.969 |
| PM_2.5_ | 0.03 | 0.792 | -0.00863 | 0.952 | 0.0629 | 0.738 | 0.113 | 0.685 | 0.00188 | 0.996 |
|  |  |  |  |  |  |  |  |  |  |  |
| (b) continued | VLF |  | TP |  | HR |  |  |  |  |  |
|  | % change | Pr(>\|t\|) | % change | Pr(>\|t\|) | % change | Pr(>\|t\|) |  |  |  |  |
| WBGT | -0.843** | 0.00266** | -0.964*** | <0.001*** | 0.612*** | <0.001*** |  |  |  |  |
| Short sleeves | 5.92 | 0.0835 | 9.47* | 0.0111* | -1.49* | 0.0464* |  |  |  |  |
| Short pants (skirts) | 10.2 | 0.1822 | 5.56 | 0.488 | -5.19** | 0.00101** |  |  |  |  |
| Gloves | -15.4** | 0.00104** | -15.6** | 0.00190** | 0.995 | 0.364 |  |  |  |  |
| Mask | -23.3*** | <0.001*** | -12.9* | 0.0107* | 1.82 | 0.0643 |  |  |  |  |
| Waterproof shoes | 1.79 | 0.6812 | 0.763 | 0.865 | 3.01*** | <0.001*** |  |  |  |  |
| Bamboo hat | 5.89 | 0.1104 | 1.87 | 0.624 | 0.447 | 0.495 |  |  |  |  |
| Age ≥ 40 | -34.2* | 0.02536* | -41.8* | 0.0115* | -2.58 | 0.354 |  |  |  |  |
| Male | -12.9 | 0.5142 | -36.0 | 0.0653 | 1.09 | 0.734 |  |  |  |  |
| Overweight/obese | -7.91 | 0.6111 | -8.26 | 0.642 | -1.55 | 0.522 |  |  |  |  |
| PM_2.5_ | 0.0522 | 0.8242 | 0.0863 | 0.714 | -0.002 | 0.948 |  |  |  |  |
|  |  |  |  |  |  |  |  |  |  |  |
| (c) | SDNN |  | RMSSD |  | LF/HF |  | LF |  | HF |  |
|  | % change | Pr(>\|t\|) | % change | Pr(>\|t\|) | % change | Pr(>\|t\|) | % change | Pr(>\|t\|) | % change | Pr(>\|t\|) |
| WBGT | -0.763*** | <0.001*** | -0.862*** | <0.001*** | -0.436* | 0.0494* | -1.78*** | <0.001*** | -1.45*** | <0.001*** |
| Long sleeves | -4.36* | 0.0204* | -5.05* | 0.0498* | -1.46 | 0.607 | -19.5*** | <0.001*** | -18.1*** | <0.001*** |
| Long pants (skirts) | -5.44 | 0.0697 | -2.66 | 0.520 | -9.07* | 0.0370* | -3.94 | 0.605 | 5.31 | 0.591 |
| Gloves | -8.67** | 0.00101** | -10.7** | 0.00282** | 3.51 | 0.400 | -18.4** | 0.0037** | -21.1** | 0.00629** |
| Mask | -8.80*** | <0.001*** | 2.07 | 0.579 | -11.2** | <0.01** | -3.58 | 0.596 | 7.17 | 0.419 |
| Waterproof shoes | 0.263 | 0.909 | -2.07 | 0.474 | -5.13 | 0.130 | -5.23 | 0.334 | 1.98 | 0.778 |
| Bamboo hat | 2.56 | 0.197 | 0.0927 | 0.972 | -1.40 | 0.632 | 1.97 | 0.688 | 2.74 | 0.657 |
| Age ≥ 40 | -21.0* | 0.0281* | -30.9* | 0.0218* | -8.86 | 0.557 | -54.7** | <0.01** | -50.3 | 0.0539 |
| Male | -4.24 | 0.642 | -1.13 | 0.935 | -16.1 | 0.200 | -17.9 | 0.414 | -2.99 | 0.9229 |
| Overweight/obese | -15.0 | 0.181 | -36.9* | 0.0116* | 76.7** | 0.00144** | -50.6* | 0.0253* | -71.8** | 0.002** |
| PM_2.5_ | 0.0239 | 0.842 | -0.0114 | 0.936 | 0.0510 | 0.786 | 0.0988 | 0.723 | -0.00343 | 0.992 |
|  |  |  |  |  |  |  |  |  |  |  |
| (c) continued | VLF |  | TP |  | HR |  |  |  |  |  |
|  | % change | Pr(>\|t\|) | % change | Pr(>\|t\|) | % change | Pr(>\|t\|) |  |  |  |  |
| WBGT | -0.883** | 0.00171** | -1.01*** | <0.001*** | 0.614*** | <0.001*** |  |  |  |  |
| Long sleeves | -5.85 | 0.0904 | -11.8*** | <0.001*** | 1.27 | 0.104 |  |  |  |  |
| Long pants (skirts) | -8.96 | 0.101 | -5.92 | 0.316 | 3.95** | 0.00149** |  |  |  |  |
| Gloves | -15.4** | 0.00102** | -15.5** | 0.00199** | 0.849 | 0.438 |  |  |  |  |
| Mask | -22.9*** | <0.001*** | -12.2* | 0.0162* | 1.62 | 0.101 |  |  |  |  |
| Waterproof shoes | 2.73 | 0.538 | 1.80 | 0.693 | 2.96*** | <0.001*** |  |  |  |  |
| Bamboo hat | 7.11 | 0.0607 | 4.23 | 0.284 | 0.268 | 0.687 |  |  |  |  |
| Age ≥ 40 | -33.1* | 0.0316* | -40.1* | 0.0178* | -3.10 | 0.268 |  |  |  |  |
| Male | -8.63 | 0.577 | -9.47 | 0.596 | -1.33 | 0.586 |  |  |  |  |
| Overweight/obese | -12.7 | 0.522 | -36.0 | 0.0680 | 0.723 | 0.822 |  |  |  |  |
| PM_2.5_ | 0.0377 | 0.873 | 0.0711 | 0.763 | -0.0007 | 0.981 |  |  |  |  |

Note: SDNN: the standard deviation of normal-to-normal intervals, RMSSD: the root mean square of successive differences between normal heartbeats, HF: high-frequency (0.15–0.4 Hz), LF: low-frequency (0.04–0.15 Hz), VLF: very-low-frequency (0.003–0.04 Hz), TP: total power, , and HR: heart rate

Table S7 Summary of the publications with WBGT monitoring in the field for (a) farmers in the open field and (b) farmers in the greenhouse

| Targeted population | No. of the Participants | Monitoring time | Monitoring year | Location | WBGT levels | Reference |
| --- | --- | --- | --- | --- | --- | --- |
| Farmer in a vegetable field | - | September to October | 2009 | Thailand | ranged 25.2-34.6 °C | Langkulsen et al., 2010 [1] |
| Rice harvester | 124 | thrice daily, April-June | 2011 | India | 26-32 °C | Sahu et al., 2013 [2] |
| Rice cultivation farmer | 29 | January and May | 2012 | Thailand | January: Mean: 25.5 °C, ranged 19.2 to 36.7°C; May: Mean 28.2 °C, ranged 20.9 to 36.3 | Langkulsen, U and Rwodzi, D, 2020 [3] |
| Maize farmer | 396 | July to September | 2016 | Nigeria | 31.56 ± 2.19 °C between 9:00 and noon; 34.08 ± 1.54 °C between noon and 15:00 | Sadiq et al., 2019 [4] |
| Sugarcane worker | 517 | February | 2017 | Guatemala | ranged 29.5 to 32.9 °C, maximum ranged 31.7 to 36.4 °C | Butler-Dawson et al., 2019 [5] |
| Date harvesting worker in the palm grove | 59 | August to September | 2017 | Iran | 37.16 ± 3.24 °C during 11:00 am - 12:59 pm | Mohammadian et al., 2020 [6] |
| Manual sugarcane cutter | 533 burned sugarcane cutters and 716 green sugarcane cutters | Burned sugarcane (Nov. to Apr.), green sugarcane (Nov to July) | 2017-2022 | Nicaragua | ranged from <28 °C to >31 °C | Hansson et al., 2024 [7] |
| Sugarcane, corn, and plantain worker | 404 | January to May | 2018 | El Salvador and Nicaragua | 32.4 ± 1.6, 34.6 ± 3.0, and 33.5 ± 1.6 °C, respectively | Petropoulos et al., 2023 [8] |
| Pregnant agricultural and manual laborer | 92 | August 2019 to December 2020 | 2019/2020 | Gambia | 24.3 ± 3.4 °C with a maximum of 35.1 °C | Bonell et al., 2022 [9] |
| Manual coffee harvester | - | May to August | - | Brazil | 23.53 ± 0.34 °C from noon to 12:59 pm and 23.78 ± 0.56 °C from 1:00 pm to 1:59 pm, higher than those from 11:00 am to noon (22.85 ± 0.27 °C) | Ramirio et al., 2021 [10] |

(b)

| Targeted population | No. of the Participants | Monitoring time | Monitoring year | Location | WBGT levels | Reference |
| --- | --- | --- | --- | --- | --- | --- |
| Elderly greenhouse worker | 120 | July to September | 2012 | South Korea | 23.0 ± 0.71 to 30.0 ± 0.83 °C inside and 20.6 ± 1.39 to 26.5 ± 0.70 °C outside of the greenhouses | Kwon et al., 2015 [11] |
| Greenhouse worker | - | August | 2020 | South Korea | 29.1 ± 4.8 °C and 27.5 ± 3.7 °C, with maximum 42.5 °C and 40.2 °C, respectively, for inside and outside the greenhouses | Jung & Kim, 2022 [12] |

Reference list for this table

1. Langkulsen U, Vichit-Vadakan N, Taptagaporn S. Health impact of climate change on occupational health and productivity in Thailand. Glob Health Action. 2010;3. <https://doi.org/10.3402/gha.v3i0.5607>.
2. Sahu S, Sett M, Kjellstrom T. Heat Exposure, cardiovascular stress and work productivity in rice harvesters in India: Implications for a Climate Change Future. Ind Health. 2013;51(4):424-31. <https://doi.org/10.2486/indhealth.2013-0006>.
3. Langkulsen U, Rwodzi D. Extreme Weather and Climate Events and Occupational Health in Thailand. In: Akhtar R, editor. Extreme Weather Events and Human Health: International Case Studies. Cham: Springer International Publishing; 2020. p. 209-25.
4. Sadiq LS, Hashim Z, Osman M. The Impact of heat on health and productivity among maize farmers in a Tropical Climate Area. J Env Pub He. 2019;2019:1-7. <https://doi.org/10.1155/2019/9896410>.
5. Butler-Dawson J, Krisher L, Yoder H, Dally M, Sorensen C, Johnson RJ, et al. Evaluation of heat stress and cumulative incidence of acute kidney injury in sugarcane workers in Guatemala. Int Arch Occ Env Hea. 2019;92(7):977-90. <https://doi.org/10.1007/s00420-019-01426-3>.
6. Mohammadian M, Heidari H, Charkhloo E, Dehghani A. Heat stress and physiological and perceptual strains of date harvesting workers in palm groves in Jiroft. Work-a Journal of Prevention Assessment & Rehabilitation. 2020;66(3):625-36. <https://doi.org/10.3233/wor-203205>.
7. Hansson E, Jakobsson K, Glaser J, Wesseling C, Chavarria D, Lucas RA, et al. Impact of heat and a rest-shade-hydration intervention program on productivity of piece-paid industrial agricultural workers at risk of chronic kidney disease of nontraditional origin. Annals of Work Exposures and Health. 2024;68(4):366-75. <https://doi.org/10.1093/annweh/wxae007>.
8. Petropoulos ZE, Keogh SA, Jarquín E, López-Pilarte D, Velázquez JJA, García-Trabanino R, et al. Heat stress and heat strain among outdoor workers in El Salvador and Nicaragua. J Expo Sci Env Epid. 2023;33(4):622-30. <https://doi.org/10.1038/s41370-023-00537-x>.
9. Bonell A, Sonko B, Badjie J, Samateh T, Saidy T, Sosseh F, et al. Environmental heat stress on maternal physiology and fetal blood flow in pregnant subsistence farmers in The Gambia, west Africa: an observational cohort study. The Lancet Planetary Health. 2022;6(12):e968-e76. <https://doi.org/10.1016/s2542-5196(22)00242-x>.
10. Ramirio LD, Sabino PHDS, Oliveira Júnior GGD, Silva ABD. Workers’ exposure to occupational heat during manual coffee harvesting. Ciência Rural. 2021;51(11). <https://doi.org/10.1590/0103-8478cr20200556>.
11. Kwon J, Park HS, Kim SH, Lee KS. Impacts of gender, weather, and workplace differences in farm worker's gear. J. Physiol. Anthropol. 2015;34. <https://doi.org/10.1186/s40101-015-0074-2>.
12. Jung W, Kim H. Evaluation of heat stress levels inside greenhouses during summer in Korea. Int J Env Res Pub He. 2022;19(19). <https://doi.org/10.3390/ijerph191912497>.

Figure S1 A side-by-side comparison was conducted for temperature (℃) with (a) 40 HOBO UA devices and (b) 40 HOBO U12 devices, as well as for relative humidity (RH) with (c) 40 HOBO U12 devices in May 2013; the coefficient of variation (CV%) among them was (a) 1.5%, (b) 1.2%, and (c) 2.8%, respectively, indicating low variability and good consistency across units. UA: Temperature: ± 0.53°C from 0° to 50°C; Temperature ± 0.35°C from 0° to 50°C; RH ±2.5% from 10% to 90% RH from the manufacturer

Figure S2 A photo with a participant wearing the sensors waist down; it can be seen in the photo that the UA (the smaller sensor) was facing up

Figure S3(a) Regression between light intensity (lm/m^2^) and solar radiation (W/m^2^) during the monitoring period of this study (sample size = 5924, 6/30 18:30 - 7/8 9:10, 2020)

Figure S3(b) A side-by-side comparison was conducted for the light intensity (lm/m^2^) with 40 HOBO UA devices in May 2013 and the coefficient of variation (CV%) among them was 7%, indicating low variability and good consistency across units.

Figure S3(c) Regression between light intensity (lm/m^2^) and solar radiation (W/m^2^) taken from July 27 to August 1, 2014 in Central Taiwan; the linear regression line shows similar slope as the one in this study conducted in 2020 shown in Figure S3(a)

Figure S3(d) Regression between light intensity (lm/m^2^) and solar radiation (W/m^2^) taken from July 18 to 22, 2014 in Central Taiwan; the linear regression line shows similar slope as the one in this study conducted in 2020 shown in Figure S3(a)

Figure S4 Distribution of PM_2.5_ levels used in the WBGT-HRV models, data were from the closest on-site indoor or outdoor sensors in the microenvironment the participants stayed; these data were the participants’ PM_2.5_ exposure

(a)

(b)

Figure S5 Examples of nonlinear relationships between (a) activity levels and the standard deviation of normal-to-normal intervals (SDNN) and (b) time of the day and SDNN.

(a)

**(b)**

Figure S6 Summary of the (a) working conditions and (c) heat-protection measures in the workplaces obtained from the interviews of the 25 participants; the percentages within each workplace are shown in the graphs

(a)

(b)

(c)

(d)

Figure S7 WBGT (℃, 5-min average) during working or non-working hours at different microenvironments in the (a) pear orchards, (b) rice seedling field, (c) greenhouse farm, and (d) all three workplaces; N: sample size, Q: quartile, IQR: inter-quartile-range.

(a)

(b)

(c)

Figure S8 Temperature (℃, 5-min average) distribution in a diurnal cycle of all the participants in the (a) pear orchard community (sample size=12517), (b) rice seedling field (sample size=8778), and (c) greenhouse farm (sample size=17609); Q: quartile, IQR: inter-quartile-range

(a)

(b)

(c)

Figure S9 Relative humidity (%, 5-min average) distribution in a diurnal cycle of all the participants in the (a) pear orchard community (sample size=12517), (b) rice seedling field (sample size=8778), and (c) greenhouse farm (sample size=17609); Q: quartile, IQR: inter-quartile-range.

(a)

(b)

(c)

Figure S10 Solar radiation (w/m^2^, 5-min average) in a diurnal cycle of all the participants in the (a) pear orchard community (sample size=12517), (b) rice seedling field (sample size=8778), and (c) greenhouse farm (sample size=17609); Q: quartile, IQR: inter-quartile-range

(a)

(b)

(c)

Figure S11 Wind speed (m/s, 5-min average) distribution in a diurnal cycle from the on-site weather station in the (a) pear orchard community (sample size=12517), (b) rice seedling field (sample size=8778), and (c) greenhouse farm (sample size=17609); Q: quartile, IQR: inter-quartile-range.

Figure S12 WBGT exposure (℃, 5-min average) during working hours classified by gender, age, and BMI group; N: sample size, Q: quartile, IQR: inter-quartile-range.

(a)

(b)

(c)

Figure S13 activity levels of the agriculture workers in a 5-min resolution in a diurnal cycle in the (a) pear orchard community, (b) rice seedling field, and (c) greenhouse farm; Q: quartile, IQR: inter-quartile-range.
